# Gene Expression in Plant Lipid Metabolism in Arabidopsis Seedlings

**DOI:** 10.1371/journal.pone.0107372

**Published:** 2014-09-29

**Authors:** An-Shan Hsiao, Richard P. Haslam, Louise V. Michaelson, Pan Liao, Johnathan A. Napier, Mee-Len Chye

**Affiliations:** 1 School of Biological Sciences, The University of Hong Kong, Pokfulam Road, Hong Kong, China; 2 Department of Biological Chemistry and Crop Protection, Rothamsted Research, Harpenden, Hertfordshire, United Kingdom; Simon Fraser University, Canada

## Abstract

Events in plant lipid metabolism are important during seedling establishment. As it has not been experimentally verified whether lipid metabolism in 2- and 5-day-old *Arabidopsis thaliana* seedlings is diurnally-controlled, quantitative real-time PCR analysis was used to investigate the expression of target genes in acyl-lipid transfer, β-oxidation and triacylglycerol (TAG) synthesis and hydrolysis in wild-type Arabidopsis WS and Col-0. In both WS and Col-0, *ACYL-COA-BINDING PROTEIN3* (*ACBP3*), *DIACYLGLYCEROL ACYLTRANSFERASE1* (*DGAT1*) and *DGAT3* showed diurnal control in 2- and 5-day-old seedlings. Also, *COMATOSE* (*CTS*) was diurnally regulated in 2-day-old seedlings and *LONG-CHAIN ACYL-COA SYNTHETASE6* (*LACS6*) in 5-day-old seedlings in both WS and Col-0. Subsequently, the effect of CIRCADIAN CLOCK ASSOCIATED1 (CCA1) and LATE ELONGATED HYPOCOTYL (LHY) from the core clock system was examined using the *cca1lhy* mutant and *CCA1*-overexpressing (CCA1-OX) lines versus wild-type WS and Col-0, respectively. Results revealed differential gene expression in lipid metabolism between 2- and 5-day-old mutant and wild-type WS seedlings, as well as between CCA1-OX and wild-type Col-0. Of the *ACBPs*, *ACBP3* displayed the most significant changes between *cca1lhy* and WS and between CCA1-OX and Col-0, consistent with previous reports that *ACBP3* is greatly affected by light/dark cycling. Evidence of oil body retention in 4- and 5-day-old seedlings of the *cca1lhy* mutant in comparison to WS indicated the effect of *cca1lhy* on storage lipid reserve mobilization. Lipid profiling revealed differences in primary lipid metabolism, namely in TAG, fatty acid methyl ester and acyl-CoA contents amongst *cca1lhy*, CCA1-OX, and wild-type seedlings. Taken together, this study demonstrates that lipid metabolism is subject to diurnal regulation in the early stages of seedling development in Arabidopsis.

## Introduction

In plant seeds, triacylglycerol (TAG) is the major storage lipid in oil bodies and functions as a critical energy reserve during germination and seedling establishment [1]–[3]. The biosynthesis of TAG occurs in the endoplasmic reticulum (ER) *via* the Kennedy pathway, and incorporates a series of membrane-bound enzymes [4]–[7]. Diacylglycerol acyltransferase (DGAT) and phospholipid:diacylglycerol acyltransferase (PDAT) catalyze the transacylation of diacylglycerol (DAG) to produce TAG [8], [9]. In germinating Arabidopsis seeds and young seedlings, *DGAT3* is more highly expressed than *DGAT1*, *DGAT2*, and *PDAT1* [3]. During germination and early post-germinative growth, the fatty acids (FAs) released from stored TAGs are converted to sucrose (Suc), providing carbon and metabolic energy for seedling development [3], [6].

Biochemical pathways in various subcellular locations participate in storage reserve mobilization [3], [10], [11]. Oil breakdown is initiated during lipolysis when TAG in oil bodies is hydrolyzed to free FA and glycerol [12]. It has been established in Arabidopsis that two TAG lipases, encoded by *SUGAR DEPENDENT1* (*SDP1*) and *SDP1-LIKE* (*SDP1L*), are responsible for the majority of oil breakdown [13], [14]. The released free FAs and/or acyl-CoA esters enter the β-oxidation pathway and are transported across the peroxisomal membrane by PEROXISOMAL ABC TRANSPORTER1 (PXA1)/PEROXISOME DEFICIENT3 (PED3)/COMATOSE (CTS) [15]–[17]. The conversion of FAs to fatty acyl-CoAs is activated by two peroxisomal long-chain acyl-CoA synthetases (LACS6 and LACS7) [18]. The three core enzymes in the β-oxidation pathway consist of acyl-CoA oxidase (ACX), multifunctional protein (MFP), and 3-ketoacyl-CoA thiolase (KAT) [3]. ACX, which catalyzes the first step of acyl-CoA oxidation in Arabidopsis, is encoded by six genes [10], [19]. The *acx1acx2* mutant lacking medium-/long-chain ACXs shows a Suc-dependent seedling establishment phenotype [20]. Of the other β-oxidation enzymes, MFP2, which catalyzes both hydration and dehydrogenation [10], is substantially induced during postgerminative seedling development [21], whilst KAT2, which catalyzes thiolytic cleavage in the last step of β-oxidation [10], is expressed during germination [22]. The Arabidopsis *kat2* mutant is defective in storage oil breakdown and is dependent on exogenous Suc during seedling establishment [22]. After β-oxidation, acetyl-CoA is converted to either citrate for respiration, or soluble sugars through the glyoxylate cycle and gluconeogenesis to support metabolism and growth [10].

During plant lipid metabolism, lipids and their acyl-CoA derivatives are transported between different subcellular compartments [10], [23]. Acyl-CoA-binding proteins (ACBPs) are candidates for such transfer because recombinant ACBPs have been demonstrated to bind acyl-CoA esters and phospholipids *in vitro* [24]–[32]. Arabidopsis ACBPs have been shown to mediate heavy metal stress tolerance [28], [33], plant defense [34], drought tolerance [35], and freezing tolerance [26], [32], [36]. Both *ACBP1* and *ACBP2* are expressed during seedling establishment [35], [37] while ACBP3 is highly expressed in germinating seedlings [38]. Some ACBPs have been reported to display diurnal expression [30], [31], [38], [39]. In 4-week-old Arabidopsis Col-0 rosettes, the expression of *ACBP4* and *ACBP5* was higher in the light period [30], while ACBP4 and ACBP5 accumulation lagged behind, with peak expression at the end of the subjective day [39]. In contrast, *ACBP3* was induced in the dark in 4-week-old Col-0 rosettes [30], [31] and 2- to 3-week-old *ACBP3pro::GUS* transformants [38].

In Arabidopsis, the clock regulatory circuit comprises a series of interlinked transcriptional feedback loops [40], [41]. The core clock loop consists of an evening-phased pseudoresponse regulator TIMING OF CAB EXPRESSION1 (TOC1) and two morning-expressed MYB transcription factors CIRCADIAN CLOCK ASSOCIATED1 (CCA1) and LATE ELONGATED HYPOCOTYL (LHY), which are reciprocally regulated [42]. CCA1 and LHY are DNA-binding proteins suppressing *TOC1* expression by binding to its 5′-flanking region [42]–[44]. As CCA1 and LHY act synergistically [45], the *cca1lhy* double mutant [44], [46]–[49] was included in this study to address the diurnal regulation of *ACBPs* and other genes in lipid metabolism in early developing Arabidopsis seedlings. As our previous studies on diurnal control of *ACBPs* were conducted using 2- to 4-week-old rosettes [30], [31], [38], we were interested to investigate if any *ACBPs* are diurnally regulated earlier in development.

Many ACBPs are stress responsive [26], [28], [29], [32]–[36] and harmony between external environmental signals and the internal clock can improve plant fitness and survival [50], [51]. For example, CCA1 regulation of defense genes allows plants to anticipate infection at dawn and better time responses to balance growth and defense [52]. This would be pertinent to ACBP3 which has been reported to play a role in plant defense [34], [38]. Furthermore, the clock is also known to control events in primary metabolism [53]–[56], for example CCA1 affects chlorophyll synthesis and biomass production, leading to starch metabolism and growth vigour [57]. Other examples include the regulation of *CCA1* by glutamate (Glu) (and Glu-derived metabolites) and *CCA1* control of nitrogen (N)-assimilatory genes [58]. Indeed, defective clock regulation reduced starch turnover and caused irregular leaf growth during the day [59]. High throughput analysis of several circadian microarray experiments revealed that about one-third of the genes expressed in 9-day-old seedlings are influenced by the biological clock [60]. As it has not been experimentally verified whether lipid metabolism in 2- and 5-day-old seedlings is diurnally-controlled, we initiated investigations on the expression of *ACBPs* and lipid metabolism genes in wild-type WS and Col-0 versus their *cca1lhy* and CCA1-OX derivatives, and subsequently demonstrated that lipid metabolism is diurnally affected even at the early stages in seedling development.

## Materials and Methods

### Plant materials and growth conditions

Wild-type Arabidopsis (*Arabidopsis thaliana*) consisted of ecotypes WS (Cs28823) and Col-0. Arabidopsis wild-type and mutant *cca1-11 lhy-21* (Cs9380) [61] seeds were purchased from the Arabidopsis Biological Resource Center (ABRC). CCA1-OX (*35S::CCA1*) was provided by Professor E.M. Tobin [62]. Seeds of each genotype were harvested at the same time from plants grown under the same conditions in the growth chamber (16 h light, 270 µmol m^−2^ s^−1^ and 8 h dark) at 22°C. Seeds were stored in a desiccator in the dark at room temperature. For germination assays, quantitative real-time PCR (qRT-PCR) and lipid analysis, seeds were surface-sterilized and germinated in half-strength Murashige and Skoog (MS) medium (Sigma-Aldrich) containing 1% (w/v) agar with 20 mM sucrose [20], to encourage more rapid seedling growth [63]. Following 4 days of 4°C treatment in the dark, plates were incubated in the tissue culture room (12 h light, 250 µmol m^−2^ s^−1^ and 12 h dark) at 22°C. For germination assays, freshly-harvested and after-ripening (harvested 3–6 months prior to use) seeds were tested and seeds were scored as germinated when radicle protrusion occurred. For Nile Red staining, seeds were grown on 1% (w/v) water-based agar.

### qRT-PCR analysis

Seedlings germinated from after-ripening seeds (harvested 3–6 months prior to use) were used for qRT-PCR analysis. Two sets of 2- and 5-day-old seedlings from each genotype were prepared in opposing 12-h-light/12-h-dark regimes according to Baudry et al. (2010) [64]. Samples were collected from both sets. Eight time points per day were selected [65]. For each time point, 500–600 2-day-old seedlings or 30–40 5-day-old seedlings were pooled for RNA isolation.

**Figure 1:**
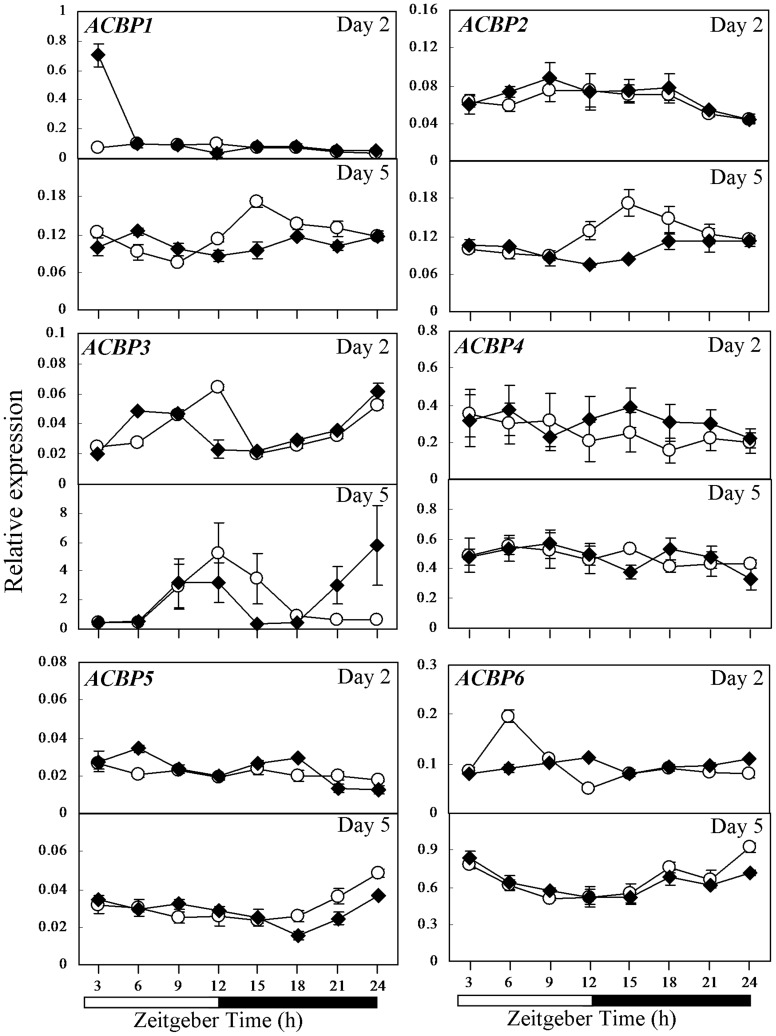
Expression pattern of the *ACBP* gene family in the *cca1lhy* mutant in comparison to wild-type WS as investigated by qRT-PCR. Expression of *ACBPs* in 2- and 5-day-old seedlings of wild-type WS (open circle) and the *cca1lhy* mutant (closed rhombus) germinated under 12-h-light/12-h-dark cycles. Relative gene expression level on the Y axis was normalized against *IPP2*. Each time point represents a mean value of six repeats from two independent biological samples ± SE. White boxes, subjective day; black boxes, subjective night.

**Figure 2:**
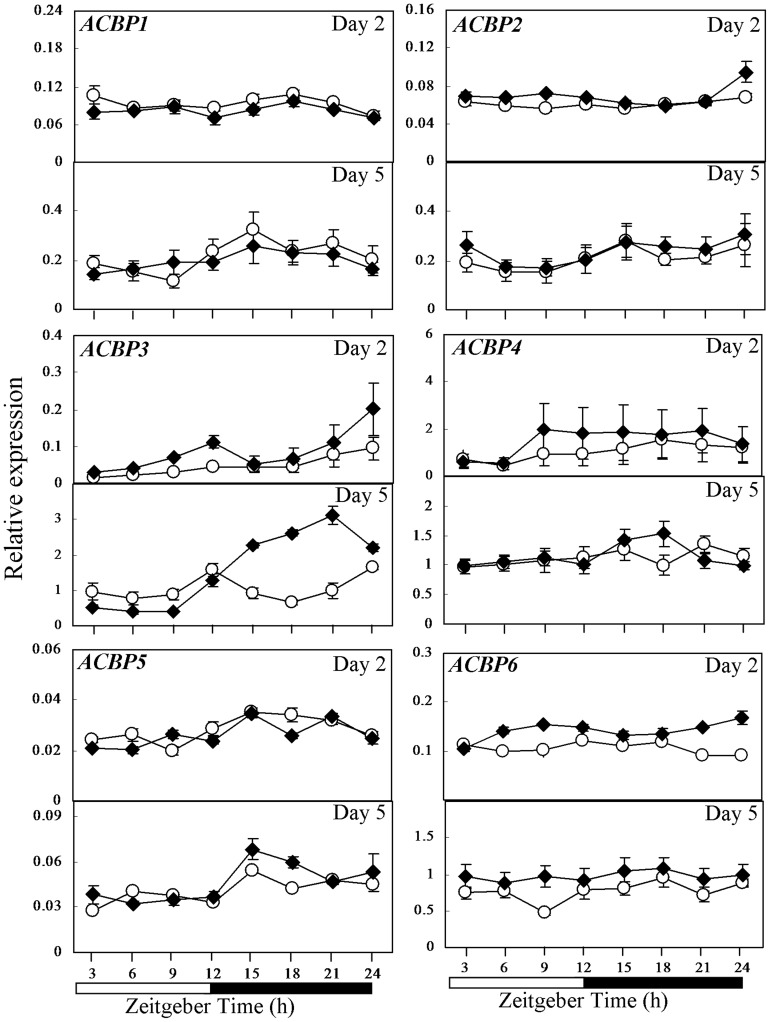
Expression pattern of the *ACBP* gene family in CCA1-OX and wild-type Col-0 as investigated by qRT-PCR. Expression of *ACBPs* in 2- and 5-day-old seedlings of wild-type Col-0 (open circle) and CCA1-OX (closed rhombus) germinated under 12-h-light/12-h-dark cycles. Relative gene expression level on the Y axis was normalized against *IPP2*. Each time point represents a mean value of six repeats from two independent biological samples ± SE. White boxes, subjective day; black boxes, subjective night.

**Figure 3:**
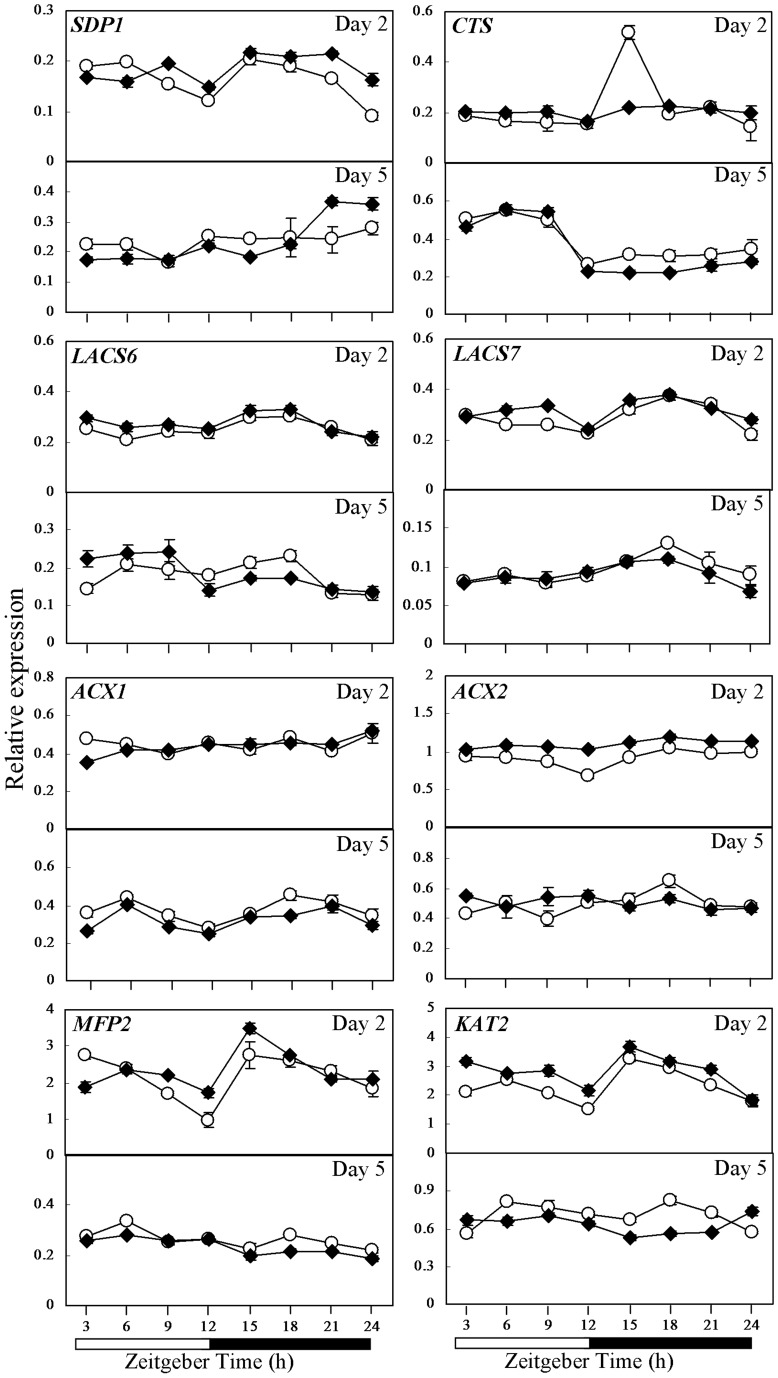
Expression pattern of genes in lipid metabolism in the *cca1lhy* mutant and wild-type WS as investigated by qRT-PCR. Expression of *SDP1*, *CTS*, *LACS6*, *LACS7*, *ACX1*, *ACX2*, *MFP2* and *KAT2* in 2- and 5-day-old seedlings of wild-type WS (open circle) and the *cca1lhy* mutant (closed rhombus) germinated under 12-h-light/12-h-dark cycles. Relative gene expression on the Y axis was normalized against *IPP2*. Each time point represents a mean value of six repeats from two independent biological samples ± SE. White boxes, subjective day; black boxes, subjective night.

**Figure 4:**
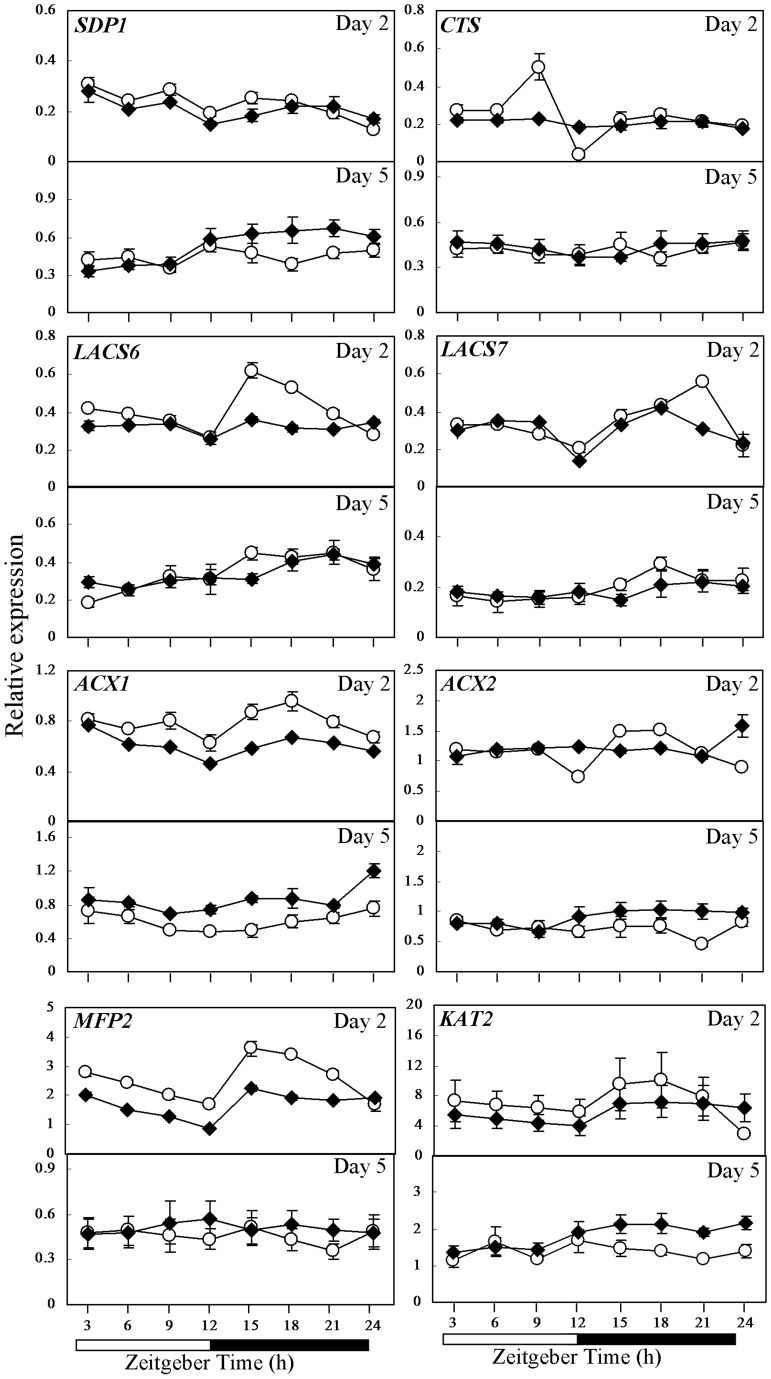
Comparison of gene expression of lipid metabolism between CCA1-OX and wild-type Col-0 as investigated by qRT-PCR. Expression of *CTS*, *SDP1, LACS6*, *LACS7*, *ACX1*, *ACX2*, *MFP2* and *KAT2* in 2- and 5-day-old seedlings of wild-type Col-0 (open circle) and CCA1-OX (closed rhombus) germinated under 12-h-light/12-h-dark cycles. Relative gene expression level on the Y axis was normalized against *IPP2*. Each time point represents a mean value of six repeats from two independent biological samples ± SE. White boxes, subjective day; black boxes, subjective night.

**Figure 5:**
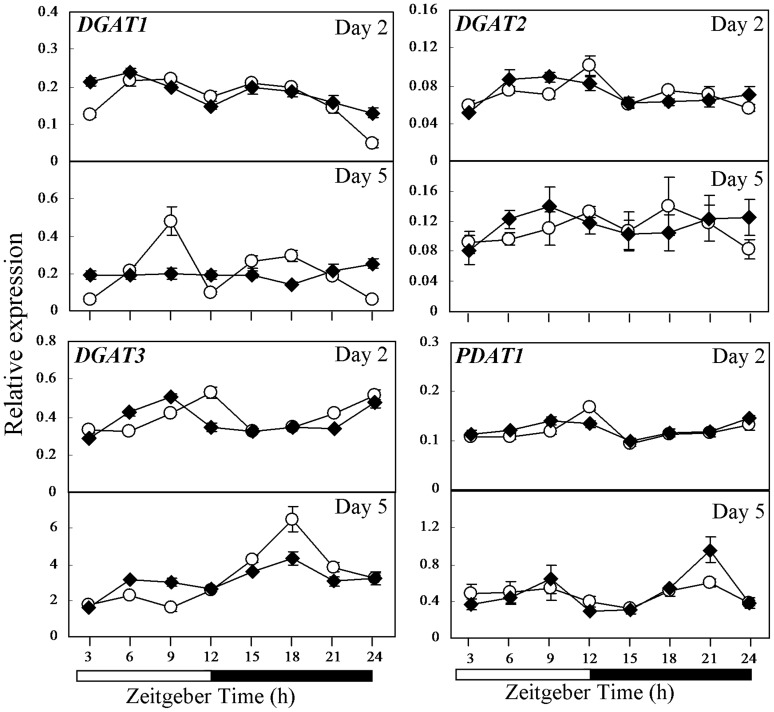
Comparison in expression of genes involved in TAG synthesis in the *cca1lhy* mutant and wild-type WS as investigated by qRT-PCR. Expression of *DGAT1*, *DGAT2*, *DGAT3* and *PDAT1* in 2- and 5-day-old seedlings of the wild type (open circle) and the *cca1lhy* mutant (closed rhombus) germinated under 12-h-light/12-h-dark cycles. Relative gene expression on the Y axis was normalized against *IPP2*. Each time point represents a mean value of six repeats from two independent biological samples ± SE. White boxes, subjective day; black boxes, subjective night.

**Figure 6:**
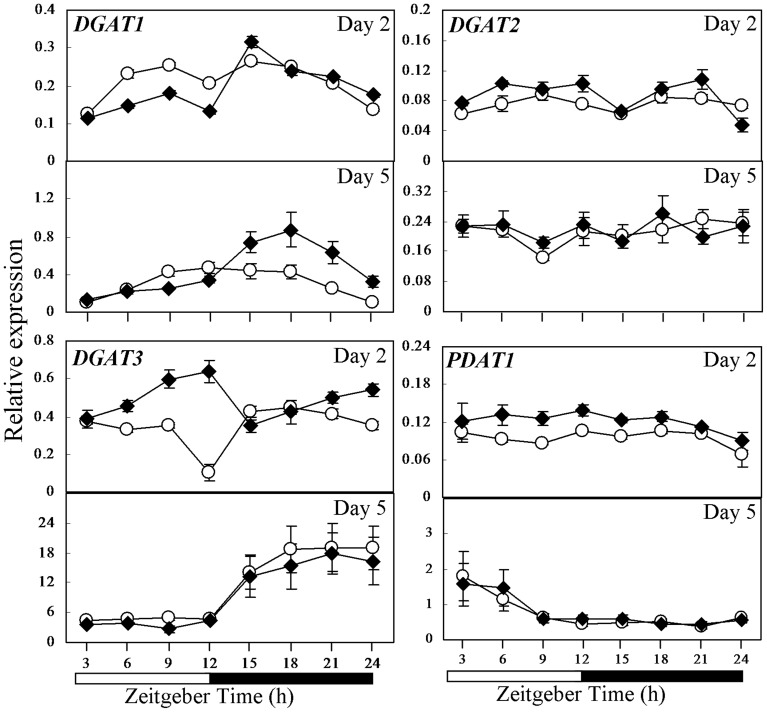
Comparison in expression of genes involved in TAG synthesis in CCA1-OX and wild-type Col-0 as investigated by qRT-PCR. Expression of *DGAT1*, *DGAT2*, *DGAT3* and *PDAT1* in 2- and 5-day-old seedlings of wild-type Col-0 (open circle) and CCA1-OX (closed rhombus) germinated under 12-h-light/12-h-dark cycles. Relative gene expression level on the Y axis was normalized against *IPP2*. Each time point represents a mean value of six repeats from two independent biological samples ± SE. White boxes, subjective day; black boxes, subjective night.

RNA, prepared using a RNeasy Isolation Kit (Qiagen), was treated with DNase and reverse-transcribed to cDNA according to the procedure supplied by the cDNA Synthesis Kit (Invitrogen). The expression of *IPP2* (isopentenyl pyrophosphate:dimethylallyl pyrophosphate isomerase 2), which is not affected by diurnal or circadian regulation in Arabidopsis seedlings, was used as an internal control [64], [66]. The expression of genes in lipid metabolism was detected in qRT-PCR using gene-specific primers as listed in Table S1. StepOne Plus (Applied Biosystems) and FastStart Universal SYBR Green Master (Roche) were utilized in qRT-PCR. Conditions for qRT-PCR were: 95°C, 10 min, followed by 40 cycles of 95°C, 15 s and 60°C, 1 min. The relative ratio of threshold cycle (C_t_) values between the *IPP2* gene and the specific gene was calculated. For quantification to calculate 2^△Ct^, three technical replicates at each time point were used. Data in Figures 1–6 and S1 represents a mean value of six repeats from two independent biological samples. Genes which displayed a 2-fold or greater value at peak expression over its lowest expression level in wild-type WS or Col-0, in both two biological repeats, were deemed to be diurnally regulated.

### Confocal microscopy

One-day-old imbibed seeds and seedlings (aged 2 to 5 days) grown under 12-h-light/12-h-dark cycles were infiltrated with an aqueous solution of Nile Red (Sigma) to visually detect neutral lipids [23], [67]–[70]. Images were obtained with a 63 X oil objective by confocal laser scanning microscopy using a Zeiss LSM 710 system equipped with argon and HeNe lasers as excitation sources. Fluorescence was excited at 514 nm and collected with a 539–653 nm filter.

### TAG extraction and mass spectrometry (MS) profiling

Dry seeds and seedlings germinated from after-ripening seeds (harvested 3–6 months prior to use) were used for lipid analysis. Dry seeds and 1- to 5-day-old seedlings germinated under 12-h-light/12-h-dark cycles were collected for lipid analysis. Seed TAGs were extracted following Bligh and Dyer (1959) [71]; seeds were heated for 10 min at 95°C in 1 ml of isopropanol and homogenized using a mortar and pestle. The homogenate was centrifuged, supernatant collected, and the pellet re-extracted. The molecular species of TAGs were analysed by electrospray ionisation triple quadrupole mass spectrometry (API 4000 QTRAP; Applied Biosystems). The profiling samples were prepared by combining 50 µl of the total lipid extract with 950 µl of isopropanol/methanol/50 mM ammonium acetate/dichloromethane (4∶3∶2∶1). TAGs [M+NH_4_]^+^ were measured following Li et al. (2014) [72] and were defined by the presence of one acyl fragment and the mass/charge of the ion formed from the intact lipid (neutral loss profiling). This allows identification of TAG acyl species and the total acyl carbons and total number of acyl double bonds in the other two chains. TAGs were quantified after background subtraction, smoothing, integration, isotope deconvolution and comparison of sample peaks with those of the internal standard (using LipidView, Applied Biosystems). The mass spectral responses of various TAG species are variable, owing to differential ionization of individual molecular TAG species. The data were normalized to the internal standard tri-17∶0 (Nu-Check Prep, USA). Fatty acid methyl esters (FAMEs) were obtained by transmethylation [73] and analyzed by gas chromatography-flame ionization detector (GC-FID) [74].

### Acyl-CoA profiling

Five-day-old seedlings germinated under 12-h-light/12-h-dark cycles were used [3]. Samples were extracted for acyl-CoA profiling according to Larson and Graham (2001) [75]. Analysis by liquid chromatography-tandem MS with multiple reaction monitoring, operated in a positive mode, was carried out [76]. Liquid chromatography separation was conducted using an Agilent 1200 LC system as previously described [77].

### Accession numbers

Sequence data included herein can be found in the Arabidopsis Genome Initiative or GenBank databases under the following accession numbers: *TOC1* (AT5G61380; NM_125531), *GI* (AT1G22770; NM_102124), *ACBP1* (AT5G53470; NM_124726), *ACBP2* (AT4G27780; NM_118916), *ACBP3* (AT4G24230; NM_118556), *ACBP4* (AT3G05420; NM_111415), *ACBP5* (AT5G27630; NM_122645), *ACBP6* (AT1G31812; NM_102916), *SDP1* (AT5G04040; NM_120486), *CTS* (AT4G39850; NM_120148), *LACS6* (AT3G05970; NM_111471), *LACS7* (AT5G27600; NM_122642), *ACX1* (AT4G16760; NM_117778), *ACX2* (AT5G65110; NM_125910), *MFP2* (AT3G06860; NM_111566), *KAT2* (AT2G33150; NM_128874), *DGAT1* (AT2G19450; NM_127503), *DGAT2* (AT3G51520; NM_115011), *DGAT3* (AT1G48300; NM_103727), *PDAT1* (AT5G13640; NM_121367), *IPP2* (AT2G39800; NM_111146).

## Results

### Analysis of the expression of *ACBPs* and other genes in 2- and 5-day-old seedlings

Two- and 5-day-old seedlings of the *cca1lhy* mutant, CCA1-OX and their respective wild types (WS and Col-0) grown in 12-h-light/12-h-dark cycles were subjected to qRT-PCR to compare the expression of *ACBPs* (Figures 1–2) and other genes in lipid metabolism, particularly target genes associated with lipolysis (*SDP1* in Figures 3–4), β-oxidation (*CTS*, *LACS6*, *LACS7*, *ACX1*, *ACX2*, *MFP2* and *KAT2* in Figures 3–4) and TAG synthesis (*DGAT1*, *DGAT2*, *DGAT3* and *PDAT1* in Figures 5–6). The relative expression of *ACBPs*, *SDP1*, *CTS*, *LACS6*, *LACS7*, *ACX1*, *ACX2*, *MFP2*, *KAT2*, *DGAT1*, *DGAT2*, *DGAT3* and *PDAT1* between the *cca1lhy* mutant and wild-type WS (Figures 1, 3, and 5) and between CCA1-OX and wild-type Col-0 (Figures 2, 4, and 6) were compared. The expression of *IPP2* (isopentenyl pyrophosphate:dimethylallyl pyrophosphate isomerase 2, At3g02780), which is known not to be influenced by diurnal or circadian control in Arabidopsis seedlings, was used as an internal control [64], [66]. As a positive control, the expression of core clock genes *TOC1* and *GIGANTEA* (*GI*) between the *cca1lhy* mutant and wild-type WS (Figure S1A) and between CCA1-OX and wild-type Col-0 (Figure S1B) were compared. *TOC1* showed peak expression at Zeitgeber time 9 (ZT9) in 2-day-old Col-0 seedlings (P<0.05; Student's *t* test), ZT12 in 2-day-old WS and 5-day-old Col-0 seedlings (P<0.05; Student's *t* test), and ZT18 in 5-day-old WS seedlings (P<0.05; Student's *t* test) (Figure S1). Peak *GI* expression occurred during the subjective day, at ZT6 in 2- and 5-day-old WS (P<0.01; Student's *t* test) and 2-day-old Col-0 seedlings and at ZT9 in 5-day-old Col-0 seedlings (P<0.01; Student's *t* test) (Figure S1). Fluctuations in expression of *TOC1* and *GI* in the wild types were generally not apparent in the *cca1lhy* mutant and CCA1-OX (Figure S1), suggesting that both the *cca1lhy* mutant and CCA1-OX are arrhythmic lines as previous reported [44], [62].

In 2-day-old WS seedlings, obvious fluctuation in expression was not observed in both *ACBP1* and *ACBP2* except at ZT3, when *ACBP1* peaked in *cca1lhy* (P<0.05; Student's *t* test) (Figure 1). In 5-day-old seedlings, both *ACBP1* and *ACBP2* mRNAs peaked in WS at ZT15 while this pattern was not evident in *cca1lhy* (P<0.05; Student's *t* test) (Figure 1). *ACBP3* expression peaked at ZT12 in 2- and 5-day-old WS and at ZT24 in 2- and 5-day-old *cca1lhy* (P<0.05; Student's *t* test) (Figure 1). In 2-day-old but not 5-day-old *cca1lhy*, *ACBP4* mRNA generally showed higher expression than WS (P<0.05; Student's *t* test) (Figure 1). In 2-day-old seedlings, *ACBP5* expression was higher at ZT6 in *cca1lhy* than in WS (P<0.05; Student's *t* test) (Figure 1). In 5-day-old WS seedlings, *ACBP5* showed higher expression than the *cca1lhy* mutant from ZT18 to ZT24 (P<0.05; Student's *t* test) but differences between them were not significant for *ACBP6* (Figure 1). In 2-day-old seedlings, *ACBP6* expression peaked at ZT6 and showed lowest expression at ZT12 in WS (P<0.001; Student's *t* test); such fluctuation was absent in *cca1lhy* (Figure 1).

Similar to WS, wild-type Col-0 showed more obvious fluctuation in *ACBP1* and *ACBP2* expression in 5-day-old rather than 2-day-old seedlings (Figure 2), again peaking at ZT15 at day 5 (Figure 1), while the expression pattern of *ACBP1* and *ACBP2* in CCA1-OX was rather similar to the wild type (Figure 2). In comparison to Col-0, peak *ACBP3* expression at ZT12 and ZT24 was greater in 2-day-old CCA1-OX (P<0.05; Student's *t* test) (Figure 2). In 5-day-old CCA1-OX, *ACBP3* showed lower expression than Col-0 from ZT3 to ZT9 but expression significantly increased between ZT15 to ZT24 (P<0.05; Student's *t* test) (Figure 2). In 2- and 5-day-old Col-0, *ACBP4* did not show obvious diurnal regulation, while in CCA1-OX its expression deviated from Col-0 with greatest differences between them at ZT9 on day 2 (P<0.05; Student's *t* test; Figure 2). In 2-day-old Col-0, both *ACBP5* and *ACBP6* mRNAs did not show obvious diurnal expression, while *ACBP5* expression was enhanced in 5-day-old CCA1-OX at ZT15 and *ACBP6* generally showed higher expression in 2- and 5-day-old CCA1-OX (P<0.05; Student's *t* test) in comparison to Col-0 (Figure 2).

### Comparative gene expression in lipid metabolism between *cca1lhy* and wild-type seedlings

When the expression of genes in lipolysis and β-oxidation in 2- and 5-day-old seedlings were analyzed by qRT-PCR, *SDP1* was down-regulated at ZT3 and ZT6, and up-regulated at ZT21 and ZT24 in *cca1lhy* versus WS (Figure 3). In *cca1lhy*, *CTS* showed lower expression than WS at ZT15 (P<0.001; Student's *t* test) especially in 2-day-old seedlings (Figure 3), but *LACS6* did not demonstrate obvious changes. However, in 2-day-old *cca1lhy*, *LACS7* showed higher expression than WS at ZT6 and ZT9 (P<0.05; Student's *t* test), but lower expression (at ZT18-24) in 5-day-old seedlings (Figure 3). *ACX1* mRNA expression was somewhat reduced in *cca1lhy* in comparison to WS especially at ZT3 (P<0.01; Student's *t* test) at days 2 and 5 (Figure 3). In 2-day-old WS, *ACX2*, *MFP2* and *KAT2* mRNAs all showed the lowest expression at ZT12 (Figure 3). However, the expression of *ACX2* was higher in 2-day-old *cca1lhy* at most time points in comparison to WS (Figure 3). *MFP2* and *KAT2* expression was generally lower (P<0.05; Student's *t* test) in 5-day-old *cca1lhy* at most time points in comparison to WS, although most *MFP2* and *KAT2* values were higher at day 2 (P<0.05; Student's *t* test; Figure 3). Hence, the genes involved in storage reserve mobilization, such as *ACX2*, *MFP2*, and *KAT2* seemed more highly-expressed in *cca1lhy* than WS at day 2 (Figure 3). At day 5, *ACX2*, *MFP2*, and *KAT2* were generally down-regulated during the subjective night in *cca1lhy* in comparison to WS (Figure 3).

Given that TAG synthesis plays a role during seedling establishment [3], *DGAT1*, *DGAT2*, *DGAT3* and *PDAT1* expression was analyzed in 2- and 5-day-old seedlings (Figure 5). Differences in *DGAT1*, *DGAT3* and *PDAT1* expression were observed between the 2- and/or 5-day-old *cca1lhy* and WS (Figure 5). Variation of *DGAT1* expression was higher in 2-day-old WS than in *cca1lhy*, with the lowest expression at ZT24 (P<0.01; Student's *t* test; Figure 5). *DGAT1* showed pronounced fluctuation in 5-day-old WS but not *cca1lhy* (Figure 5). In contrast, *DGAT1* and *DGAT3* expression in 5-day-old *cca1lhy* seemed less affected than WS by light/dark cycling whilst there was no significant difference between the genotypes in *DGAT2* expression in both 2- and 5-day-old seedlings (Figure 5). Five-day-old WS showed peak *DGAT1* expression at ZT9 with a second peak at ZT18 (Figure 5). Peak *DGAT3* expression occurred at ZT18 (Figure 5). Absence of these peaks in the mutant suggests that diurnal control of *DGAT1* and *DGAT3* was quashed. Interestingly, *PDAT1* expression showed greater fluctuation in 5-day-old *cca1lhy* than WS but such differences were less obvious in 2-day-old seedlings (Figure 5). These findings provide evidence for diurnal control of TAG synthesis in *DGAT1*, *DGAT3*, and *PDAT1* expression in 2- and 5-day-old seedlings.

### Comparative gene expression in lipid metabolism between CCA1-OX and wild-type seedlings

To further investigate the role of *CCA1* in lipid metabolism, the expression of *CTS*, *SDP1, LACS6*, *LACS7*, *ACX1*, *ACX2*, *MFP2* and *KAT2* in 2- and 5-day-old seedlings germinated under 12-h-light/12-h-dark cycles of wild-type Col-0 and CCA1-OX was analyzed by qRT-PCR (Figure 4). In 2-day-old Col-0, *CTS* and *LACS7* expression peaked at ZT9 and ZT21, respectively, while *LACS6* peaked at ZT15 and the lowest expression of *SDP1*, *CTS*, *LACS6*, *LACS7*, *ACX1*, *ACX2*, *MFP2* and *KAT2* appeared at ZT12 or ZT24 (Figure 4). Genes involved in storage reserve mobilization in CCA1-OX were generally down-regulated in 2-day-old seedlings and up-regulated in 5-day-old seedlings in comparison to Col-0 (Figure 4). In particular, in 2-day-old CCA1-OX, *SDP1* expression was generally lower than Col-0 from ZT6 to ZT15, *LACS6* from ZT15 to ZT21, *ACX1* from ZT15 to ZT24, *MFP2* from ZT3 to ZT21 and *KAT2* from ZT3 to ZT21 (Figure 4). Also, loss in diurnal regulation was evident in 2-day-old CCA1-OX for *CTS*, in contrast to Col-0 which peaked at ZT9 and showed lowest expression at ZT12 (P<0.05; Student's *t* test) (Figure 4). Other deviations from wild-type diurnal control in 2-day-old CCA1-OX were observed for *LACS7* and *ACX2* (Figure 4).

In 5-day-old seedlings, *SDP1* mRNA in CCA1-OX was significantly up-regulated from ZT15 to ZT24 (P<0.05; Student's *t* test; Figure 4). Similar to *SDP1*, *ACX2* (P<0.05; Student's *t* test) and *KAT2* (P<0.01; Student's *t* test) were more highly expressed in 5-day-old CCA1-OX during the subjective night while *ACX1* showed higher expression at most time points (P<0.05; Student's *t* test; Figure 4). These results suggest that some genes in lipid metabolism are diurnally-regulated during germination and seedling establishment as captured on 2- and 5-day-old seedlings.

In wild-type Col-0, *DGAT1* showed lowest expression at ZT3 and ZT24 at both days 2 and 5; *DGAT3* showed lowest expression at ZT12 at day 2 but was generally induced in the subjective night at day 5; and *PDAT1* showed highest expression at ZT3 on day 5 (Figure 6). When the expression of *DGAT1*, *DGAT2*, *DGAT3*, and *PDAT1* in 2- and 5-day-old CCA1-OX was compared to Col-0, there were no clear changes in *DGAT2* expression. The expression of *DGAT1* in CCA1-OX appeared induced in the subjective night, peaking at ZT15 (P<0.05; Student's *t* test) in 2-day-old seedlings (Figure 6). In contrast, the expression of *DGAT3* was generally higher than Col-0 in the subjective day at 2 days (P<0.05; Student's *t* test; Figure 6). *PDAT1* expression in 2-day-old CCA1-OX was generally higher (P<0.05; Student's *t* test) than Col-0 but this pattern was not retained in 5-day-old seedlings (Figure 6). In summary, when CCA1 was overexpressed in Col-0, *DGAT1* and *DGAT3* were more affected than *DGAT2* and *PDAT1* in both 2- and 5-day-old seedlings (Figure 6).

### Data mining of microarray analysis specific to 9-day-old seedlings

Covington et al. (2008) [60] have integrated information from multiple circadian microarray experiments using 9-day-old Arabidopsis seedlings to evaluate the circadian transcriptome. Genes that were expressed (Exp) and those under circadian control (Cir) are summarized (Table S2) in a total of nine datasets, including four original datasets: namely Covington [78], Edwards [79], Michael 1 and Michael 2 [55]; three of which the original Covington (C) and Edwards (E) datasets were combined in three different ways: CECE, CCEE, and EECC; and finally two combined datasets: C+E intersection and C+E union [60]. Mining their summarized data (Additional Data File 2 in [60]), we identified *ACBPs* and the genes associated with lipolysis, β-oxidation and TAG synthesis that were expressed, as well as those that showed clock regulation (Table S2), and the expression pattern of the selected genes in the normalized CCEE dataset is presented in Figure S2. *ACBPs* did not show any obvious clock regulation in 9-day-old seedlings (Table S2) while *ACBP2* and *ACBP5* showed some fluctuations (Figure S2). The lipolysis gene *SDP1* demonstrated clock regulation in all nine datasets (Table S2), peaking at the subjective day (Figure S2). Of the β-oxidation genes, *CTS* indicated clock regulation only in the Covington dataset (Table S2); while *LACS6* displayed clock regulation in seven datasets except for the original Covington and Edwards datasets (Table S2), peaking at the late subjective day (Figure S2). *LACS7* and *MFP2* did not exhibit any evidence of clock regulation in all nine datasets (Table S2). *ACX1*, peaking at the subjective night or from the late subjective night to the early subjective day (Figure S2) and *KAT2*, peaking from the late subjective day to the early subjective night (Figure S2), showed clock regulation in most datasets except in the original Edwards and Covington datasets, respectively (Table S2); whilst *ACX2* demonstrated such regulation only in Michael 1 and Michael 2 (Table S2). Of the TAG synthesis genes, *DGAT1* (but not *DGAT2*) showed clock regulation in all nine datasets (Table S2), peaking at the late subjective day (Figure S2). *PDAT1* was clock-regulated in six datasets except the original Covington, Michael 1, and Michael 2 (Table S2), peaking at the subjective day (Figure S2). In general, genes under clock control showed high expression in the subjective day or at day-night transition.

### The *cca1lhy* mutant retains oil bodies

**Figure 7:**
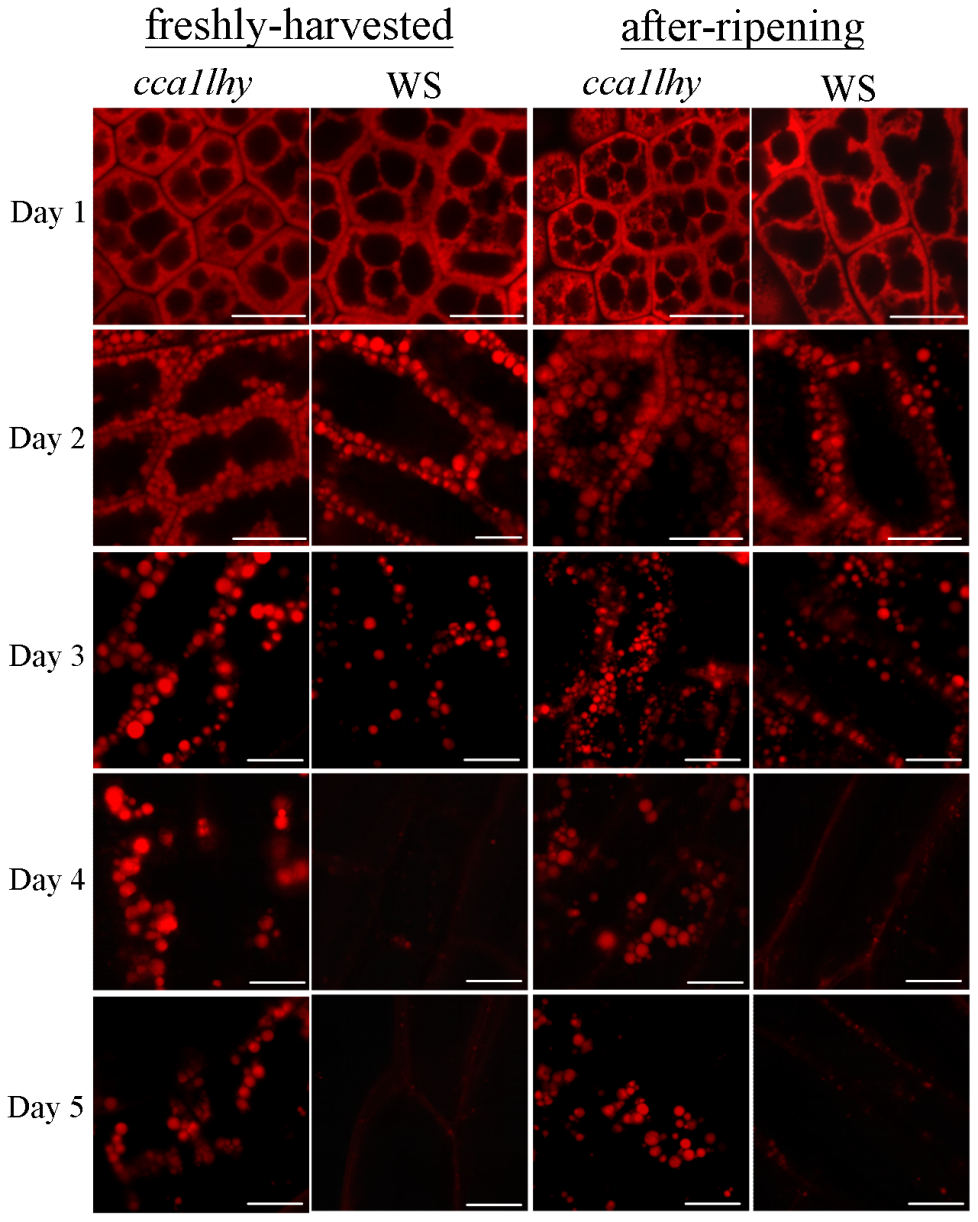
The *cca1lhy* mutant showed oil body retention in comparison to wild-type WS. Confocal laser microscopy of Nile Red stained oil bodies of the radicle from 1-day-old imbibed seeds (Day 1) and hypocotyl epidermis from 2- to 5-day-old seedlings (Day 2 to Day 5) of the *cca1lhy* mutant and wild-type WS germinated under 12-h-light/12-h-dark cycles on water-based agar. Samples from freshly-harvested and after-ripening seeds of the *cca1lhy* mutant and wild-type WS are shown. Scale bar  =  10 µm.

It has been reported that oil body accumulation is a phenotype observed in Arabidopsis mutants abrogated in the mobilization of storage reserves [13], [16], [18], [20]–[22]. Freshly-harvested and after-ripening seeds of *cca1lhy* and WS, germinated under 12-h-light/12-h-dark cycles on water-based agar, were stained with Nile Red during seedling growth from days 1 to 5 after imbibition (Figure 7). Confocal laser microscopy revealed the presence of red-stained spherical inclusions representing oil body accumulation in *cca1lhy* from days 1 to 5 in comparison to WS (Figure 7). In *cca1lhy*, the oil bodies were evident at day 5 in samples derived from freshly-harvested and after-ripening seeds (Figure 7) although seedling establishment would have been completed by then [20]. In WS, the number of oil bodies had substantially declined by day 3 and very few were evident by days 4 and 5 (Figure 7). When the germination frequency was investigated using freshly-harvested seeds of the *cca1lhy* mutant and wild-type WS cultured under 12-h-light/12-h-dark cycles on half-strength MS medium containing 20 mM sucrose, there were no significant differences between them except at day 1 when the wild type germinated better (Figure S3A). However, after-ripening (Figure S3B) seeds of the *cca1lhy* mutant were slightly impaired in germination in comparison to the wild type over a 7-day observation period.

### TAG accumulates in 5-day-old *cca1lhy* seedlings

**Figure 8:**
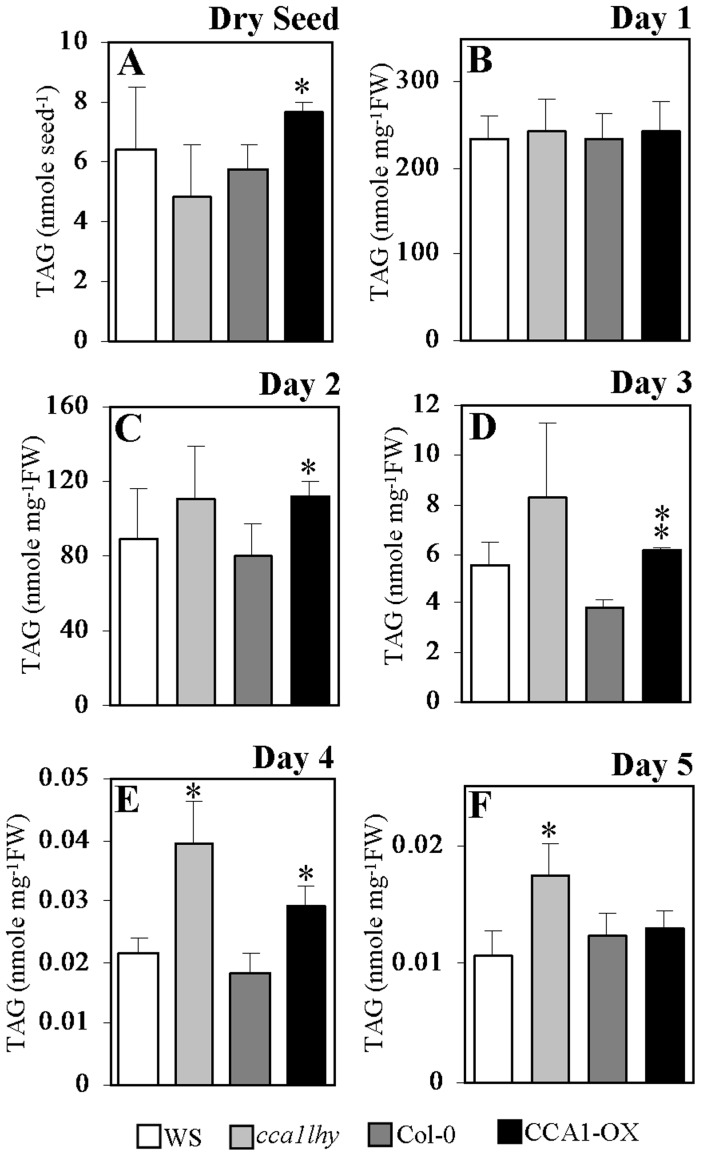
Dry seed and seedling TAG content of the wild types, the *cca1lhy* mutant and CCA1-OX during germination and early post-germinative growth. (A) Total TAG content of wild-type, *cca1lhy*, and CCA1-OX dry seeds. (B)–(F) Total TAG content of wild-type, *cca1lhy*, and CCA1-OX seedlings at Day 1 (B), Day 2 (C), Day 3 (D), Day 4 (E), and Day 5 (F) cultured under 12-h-light/12-h-dark cycles on half-strength MS medium supplemented with 20 mM sucrose. White bar, wild-type WS; light gray bar, the *cca1lhy* mutant; dark gray bar, wild-type Col-0; black bar, CCA1-OX. Data in (A) represents a mean value of three repeats ± SD per seed, each measurement contains 20 dry seeds. Data in (B)–(F) represents a mean value of three repeats ± SD per mg fresh weight (FW) of seedlings, per measurement contains 96–122 mg seedlings. Student's *t* test for *, P<0.05; **, P<0.01.

When 4- and 5-day-old *cca1lhy* seedlings showed oil body accumulation in contrast to the wild type (Figure 7), we were prompted to investigate whether this coincided with changes in TAG content. Dry seed and seedling samples collected at regular intervals, 1 to 5 days after imbibition under 12-h-light/12-h-dark cycles, were analyzed for TAG content by electrospray ionization–tandem mass spectrometry mass spectrometry (ESI-MS/MS) (Figure 8). CCA1-OX dry seeds showed significantly higher TAG content than wild-type Col-0 (Figure 8A), while *cca1lhy* seemed to contain slightly lower TAG than WS (Figure 8A), but this difference was not statistically significant (Figure 8A). At day 1 after imbibition, there were no apparent differences in TAG content amongst *cca1lhy*, CCA1-OX, and their respective wild types (Figure 8B). At days 2 and 3 after imbibition, only CCA1-OX indicated an elevated TAG content in comparison to Col-0 (Figure 8C–D), and this was maintained to day 4 (Figure 8E). The increase of TAG in *cca1lhy* over WS appeared at days 4 and 5 after imbibition (Figure 8E–F). The *cca1lhy* mutant showed highest TAG content amongst all the genotypes on days 4 to 5 (Figure 8E–F). In contrast, by day 5 there were no apparent differences between CCA1-OX and Col-0 (Figure 8F).

### Total FAs accumulate in 5-day-old *cca1lhy* seedlings

**Figure 9:**
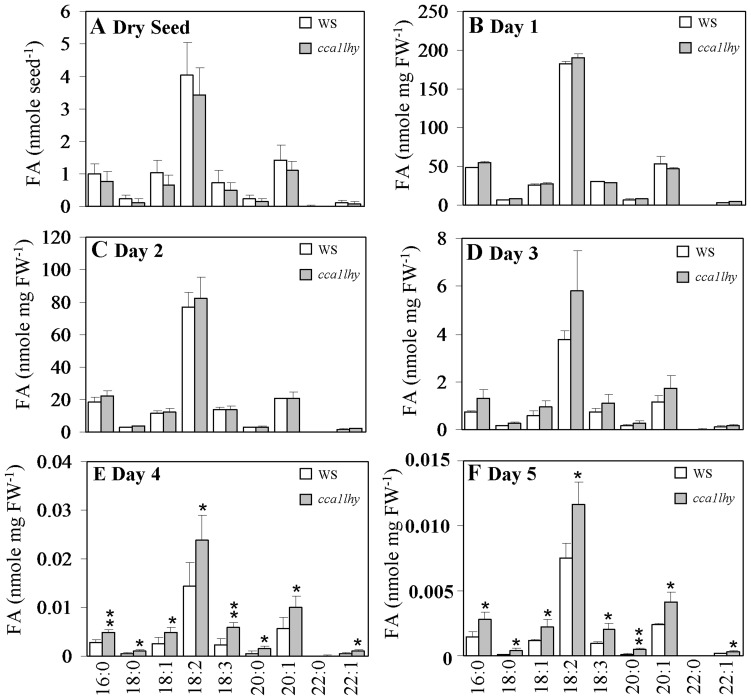
Fatty acid profiling of WS and *cca1lhy* seeds and seedlings during germination and early post-germinative growth. (A) Major fatty acid (FA) content of WS and *cca1lhy* dry seeds. (B)–(F) Major FA content of WS and *cca1lhy* seedlings at Day 1 (B), Day 2 (C), Day 3 (D), Day 4 (E), and Day 5 (F) cultured under 12-h-light/12-h-dark cycles on half-strength MS medium supplemented with 20 mM sucrose. White bar, wild-type WS; light gray bar, the *cca1lhy* mutant. Data in (A) represents a mean value of three repeats ± SD per seed, each measurement contains 20 dry seeds. Data in (B)–(F) represents a mean value of three repeats ± SD per mg fresh weight (FW) of seedlings, per measurement contains 96–122 mg seedlings. Student's *t* test for *, P<0.05; **, P<0.01.

**Figure 10:**
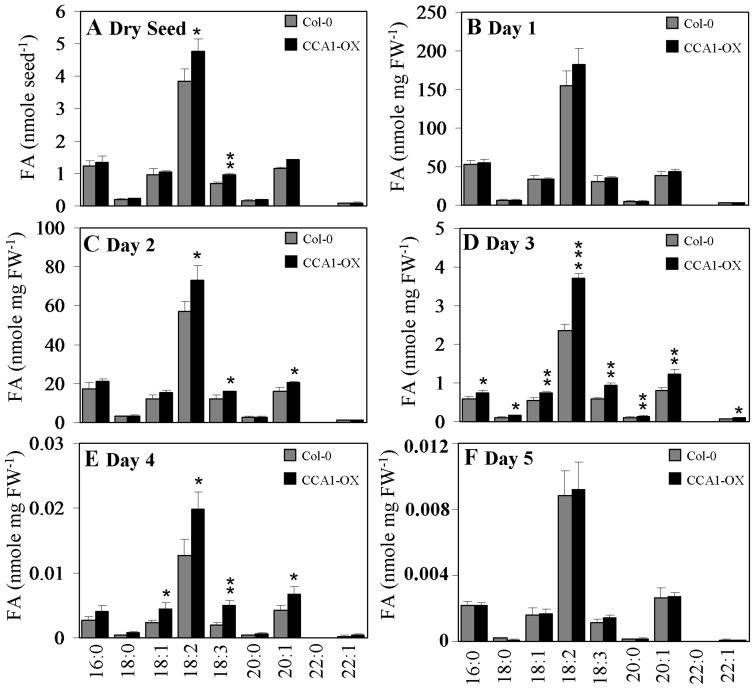
Fatty acid profiling of Col-0 and CCA1-OX seeds and seedlings during germination and early post-germinative growth. (A) Major fatty acid (FA) content of Col-0 and CCA1-OX dry seeds. (B)-(F) Major FA content of Col-0 and CCA1-OX seedlings in Day 1 (B), Day 2 (C), Day 3 (D), Day 4 (E), and Day 5 (F) cultured under 12-h-light/12-h-dark cycles on half-strength MS medium supplemented with 20 mM sucrose. Dark gray bar, wild-type Col-0; black bar, CCA1-OX. Data in (A) represents a mean value of three repeats ± SD per seed, each measurement contains 20 dry seeds. Data in (B)–(F) represents a mean value of three repeats ± SD per mg fresh weight (FW) of seedlings, per measurement contains 96–117 mg seedlings. Student's *t* test for *, P<0.05; **, P<0.01.

Changes in FA composition were identified between *cca1lhy* and WS (Figure 9), as well as CCA1-OX and Col-0 (Figure 10), using GC-FID analysis of FAMEs on dry seeds and seedlings grown under 12-h-light/12-h-dark cycles. In dry seeds and 1- to 3-day-old seedlings, there were no significant changes in fatty acid content between *cca1lhy* and WS (Figure 9A–D). However, *cca1lhy* showed an increase in fatty acids at days 4 to 5 after imbibition in comparison to WS (Figure 9E–F).

Interestingly, in comparison to Col-0, CCA1-OX showed some compositional changes i.e. higher 18∶2 and 18∶3 fatty acid content in dry seeds (Figure 10A), similar fatty acid content in 1-day-old seedlings (Figure 10B), higher 18∶2, 18∶3, and 20∶1 fatty acid content in 2-day-old seedlings (Figure 10C), increased 16∶0, 18∶0, 18∶1, 18∶2, 18∶3, 20∶0, 20∶1, and 22∶1 fatty acid content in 3-day-old seedlings (Figure 10D), higher 18∶1, 18∶2, 18∶3, and 20∶1 fatty acid content in 4-day-old seedlings (Figure 10E), but similar fatty acid content in 5-day-old seedlings (Figure 10F).

### Acyl-CoA profiling highlighted differences in acyl-CoA composition between *cca1lhy*, CCA1-OX, and wild-type seedlings

During storage reserve mobilization in post-germination development, fatty acids from oil bodies are activated to acyl-CoAs before they enter peroxisomal FA β-oxidation [19]. To determine the acyl-CoA compositional changes amongst *cca1lhy*, CCA1-OX, and wild-type Arabidopsis, the acyl-CoA pool was profiled in 5-day-old seedlings germinated under 12-h-light/12-h-dark cycles. Some acyl-CoA esters (16∶0, 16∶3, 24∶0, and 26∶0) accumulated in *cca1lhy* in comparison to wild-type WS (Figure S4); whilst others, e.g. 18∶2, 22∶1, and 28∶1-CoA, decreased in *cca1lhy* in comparison to WS (Figure S4). When compared to wild-type Col-0, 16∶3, 18∶3, 20∶0, 20∶1, 22∶1, 24∶1 and 26∶1-CoA were reduced in CCA1-OX (Figure S4). Only 24∶0 and 30∶0-CoA accumulated in CCA1-OX in comparison to Col-0 (Figure S4).

## Discussion

### Comparative expression of *ACBPs* and lipid metabolism genes in Arabidopsis seedlings

In plants, it has been reported that the mRNAs of many metabolic enzymes are clock-regulated [53]–[56]. For example, the expression of genes involved in chlorophyll biosynthesis peaked at late dark; those of the electron transport photosystems peaked in the light; starch synthesis genes were highly expressed at early light or during the day in contrast to starch degradation genes which peaked at late light; and most genes related to nitrogen and sulfate assimilation peaked at the subjective night or at early light [53], [56], [80]–[83]. Various enzymes involved in plant lipid biosynthesis including β-ketoacyl-CoA synthase 16 (KCS16; At4g34250), acyl-CoA desaturase-like 2 (ADS2; At2g31360), sphingolipid Δ8-desaturase 2 (SLD2; At2g46210), UDP-Glc:sterol glucosyltransferase (UGT80A2; At3g07020), CDP-DAG synthase 1 (CDS1; At1g62430), and lecithine cholesterol acyltransferase-like protein (At1g27480) are known to be transcriptionally regulated by the biological clock [53].

In this study, the comparative expression of *ACBPs* and lipid metabolism genes was investigated in 2- and 5-day-old Arabidopsis seedlings. Consistent with previous reports that *TOC1* peaks at ZT12 [43] and *GI* peaks during the subjective day in 7-day-old WS seedlings [84], our investigation on WS and Col-0 seedlings revealed that *TOC1* expression also peaked at ZT12 in 2-day-old WS and 5-day-old Col-0, while *GI* peaked during the subjective day in both 2- and 5-day-old WS and Col-0 (Figure S1). The expression of *ACBPs* and lipid metabolism genes was observed herein to align with the expression of *TOC1* and *GI* and they too showed variation in expression between the *cca1lhy* mutant and wild-type WS (Figure S1A) and between CCA1-OX and wild-type Col-0 (Figure S1B). At days 2 and 5, *ACBP3* showed more obvious diurnal regulation in wild-type WS (Figure 1) than Col-0 (Figure 2). In 5-day-old wild-type WS and Col-0 seedlings, a similar diurnal expression pattern was observed for *ACBP1* and *ACBP2* (Figures 1, 2 and S5). Fluctuation of *ACBP6* expression was more obvious in 2-day-old wild-type WS (Figures 1 and S5). As candidates for acyl-lipid transfer, fluctuation in expression of *ACBPs* suggests that it may diurnally affect acyl-lipid metabolism in seedlings. When we compared the expression pattern between WS and *cca1lhy*, our results revealed that the expression pattern of all *ACBP* mRNAs in *cca1lhy* slightly deviated from the wild type (Figure 1). Moreover, the diurnal expression pattern of *ACBPs* in wild-type Col-0 seedlings germinated under 12-h-light/12-h-dark cycles showed some differences from results conducted on 4-week-old rosettes under 16-h-light/8-h-dark [30]: both *ACBP1* and *ACBP2* showed peak expression at ZT15 in 5-day-old seedlings but not in rosettes; both *ACBP4* and *ACBP5* showed peak expression at ZT9 in rosettes but not in 2- and 5-day-old seedlings. Nevertheless, similarity was noted in *ACBP3* peaks at ZT24 in 2-day-old seedlings and 4-week-old rosettes. Indeed, *ACBP3* expression displayed the most contrast between the *cca1lhy* mutant and WS (Figure 1) and between CCA1-OX and Col-0 (Figure 2), consistent with our previous reports that *ACBP3* mRNA is most affected by light/dark cycling [30], [31] and that the 5′-flanking region of *ACBP3* is responsive to dark/light [38]. This finding relating ACBP3 to CCA1 supports a previous report that CCA1 regulates plant defense [52]; ACBP3 has been shown to play a role in the plant defense response [34], [38]. Furthermore, variations in the gene expression patterns of *ACBPs* in seedlings and rosettes indicate that diurnal regulation may alter at various stages in plant development.

Besides *ACBPs*, genes related to storage reserve mobilization showed diurnal fluctuation in expression in wild-type seedlings (Figures 3, 4 and S5). In some cases peak expression differed between WS and Col-0. For example in 2-day-old WS, peak expression of *CTS* was observed at ZT15 (Figure 3) in contrast to ZT9 in Col-0 (Figure 4). Nevertheless, in both 2-day-old wild-type WS and Col-0 seedlings, a similar expression pattern was observed for *SDP1* with lowest expression at ZT24 (Figures 3–4). In 2-day-old WS seedlings, lowest expression at ZT12 was noted for *ACX2*, *MFP2* and *KAT2* (Figure 3) while in 2-day-old Col-0 seedlings two troughs at ZT12 and ZT24 were observed for *LACS6*, *ACX2* and *KAT2* (Figure 4). Genes involved in storage reserve mobilization somewhat showed reduced expression at day-night transition (ZT12 and ZT24) in 2-day-old wild-type seedlings but this pattern became less obvious in older (5-day-old) seedlings (Figures 3–4). Such fluctuations suggest that β-oxidation may be subject to diurnal regulation in these young Arabidopsis seedlings (Figure S5).

Loss of diurnal regulation of *CTS* was observed in both 2-day-old *cca1lhy* and CCA1-OX seedlings (Figures 3–4). *ACX2*, *MFP2* and *KAT2* were generally up-regulated in 2-day-old *cca1lhy* and *MFP2* and *KAT2* mildly down-regulated during the subjective night at day 5 in comparison to WS (Figure 3). In contrast, *ACX1*, *MFP2* and *KAT2* were generally down-regulated in 2-day-old CCA1-OX seedlings and up-regulated at day 5 in comparison to Col-0 (Figure 4). Taken together, these results suggest that the expression of genes in storage reserve mobilization is altered in both *cca1lhy* and CCA1-OX seedlings. *ACX1*, *MFP2* and *KAT2* have corresponding mutants previously reported to show an oil body retention phenotype [20]–[22]. Such down-regulation may explain for the oil body accumulation phenotype we observed in the 4- and 5-day-old *cca1lhy* mutants (Figure 7) arising from a reduction in lipid catabolism.

Genes of TAG synthesis as well as *ACBPs* and genes involved in storage reserve mobilization showed fluctuation in expression in wild-type seedlings (Figures 5, 6 and S5). When comparing the expression pattern in the wild types versus the *cca1lhy* mutant or CCA1-OX, both *DGAT1* and *DGAT3* showed different patterns between 5-day-old *cca1lhy* and WS (Figure 5) as well as CCA1-OX and Col-0 (Figure 6), suggesting that both genes play important roles in seedling establishment and TAG recycling during storage reserve mobilization, in good agreement with the results of Hernández et al. (2012) [3]. The lack of diurnal fluctuation in the expression of *DGAT1* and *DGAT3* in 5-day-old *cca1lhy* in comparison to WS (Figure 5) and the increased fluctuation of *PDAT1* expression in *cca1lhy* (Figure 5) may also account for the significant lipid changes in TAG (Figure 8) and FAs (Figure 9) at day 5 (and day 4). In summary, the qRT-PCR results have indicated that both TAG synthesis and its hydrolysis is affected in the *cca1lhy* mutant.

Our data mined from microarray analysis [60] suggested that *SDP1*, *LACS6*, *ACX1*, *KAT2*, *DGAT1*, and *PDAT1* are circadian regulated in at least six datasets (Table S2). Our qRT-PCR is consistent with Covington et al. (2008) [60] in that the expression of some genes in lipid metabolism is diurnally-regulated. We had observed a diurnal expression pattern of target genes in WS and Col-0 and differences between 2- and 5-day-old *cca1lhy* and WS seedlings, as well as between CCA1-OX and Col-0 (Figures 3, 4, 5, 6 and S5).

However, day 2 is important and significant changes in transcription patterns of genes involved in storage reserve mobilization have been reported [63]. Therefore, variation in gene expression within day 2 might partially be ascribed to developmental rather than diurnal regulation. Moreover, for many metabolic genes, cycling of transcripts need not correlate to changes in maximal catalytic activity or protein level. For example, the mRNAs of many genes in starch degradation oscillate, but their protein levels are kept constant in Arabidopsis [80], [82], [83], [85]. Hence, we caution that our transcriptomic analysis merely provides evidence of diurnal regulation of lipid metabolism at the level of transcription.

### Comparison between the *cca1lhy* mutant and WS at germination

Except for day 1, the germination rate of freshly-harvested seeds after stratification did not significantly differ between the *cca1lhy* mutant and wild-type WS (Figure S3A), consistent with a previous report [86] which concluded that circadian clock genes coordinate environmental signalling affecting dormancy release in plants. Herein, we used 4-day stratification at 4°C to eliminate the effect of dormancy and subsequently investigated the relationship between the biological clock and lipid metabolism from germination to seedling establishment. Our data showed only minor changes from days 1 to 7 between the *cca1lhy* mutant and wild-type WS in germination rate for after-ripening seeds (Figure S3B). An oil body retention phenotype was evident in seedlings germinated from either freshly-harvested seeds or after-ripening seeds of the *cca1lhy* mutant (Figure 7), suggesting that seed germination and storage reserve mobilization are regulated independently as previously shown [87]. In after-ripening seeds, the germination frequencies between the *cca1lhy* mutant and the wild type further diversified (Figure S3B), in contrast to the hypersensitive dominant phenotype that freshly-harvested seeds of the *cca1lhy* mutant showed a higher frequency of germination than the wild type and exhibited germination hypersensitivity on cold treatments of 1, 2, and 3 days [86]. Our results indicate that dormant seed and non-dormant seed germination, though differentially regulated, are subject to diurnal control and establish a link between clock regulation and lipid metabolism in Arabidopsis seedlings.

### Lipid profiling indicates lipid metabolism is altered in *cca1lhy* and CCA1-OX seedlings

In plants, storage reserve mobilization that occurs during seed germination and early seedling establishment is known to be a dynamic process [10], [11]. A process which we demonstrate is intimately linked to internal plant biological clock. Herein, we showed an oil body accumulation phenotype in the *cca1lhy* mutant, indicating that lipid metabolism in Arabidopsis seedlings is affected by clock components (CCA1 and LHY). Direct evidence for the influence of the clock in metabolism can be obtained from measurements of metabolites in clock mutants [83]. The Arabidopsis arrhythmic mutant *prr5prr7prr9* had elevated levels of citric acid cycle intermediates and other metabolites, including amino acids [88]. With regard to lipids, the amounts of linoleic and linolenic acids showed temperature-related fluctuations in cotton seedlings [89]. It has also been shown that only 18∶1 FA, but not other fatty acids, oscillates under diurnal cycles in Arabidopsis, with higher levels accumulated in the light rather than the dark [90]. Besides FA, phosphatidylcholine was recently reported to oscillate diurnally and affects florigen-mediated flowering [91].

Previous reports have revealed that the accumulation of 20∶1-CoA is characteristic of the storage reserve mobilization related mutants, *cts*/*pxa1*, *lacs6lacs7*, *acx1acx2*, *mfp2* and *kat2* [3], [16], [18], [20]–[22]. In this study, although a slight down-regulation of various β-oxidation key enzymes including *ACX1*, *MFP2*, and *KAT2*, was observed in 5-day-old *cca1lhy* (Figure 3), 20∶1-CoA did not accumulate (Figure S4), suggesting that TAG hydrolysis may be more affected by diurnal regulation than β-oxidation. However, some acyl-CoAs (16∶0, 16∶3, 24∶0, and 26∶0) accumulated significantly in 5-day-old *cca1lhy* in comparison to WS (Figure S4), while an 18∶1-CoA increase was reported in the 5-day-old *acx1acx2*, *mfp2* and *kat2* mutants [3], [20], [22] and 18∶2-CoA accumulation in the 5-day-old *acx1acx2* mutant [3], [20]. Elevation in different kinds of acyl-CoA species might indicate variation in transport mechanisms during storage reserve mobilization. Other than β-oxidation, it has been reported that the N-end rule pathway can regulate seed oil mobilization as was proven using mutants in *PROTEOLYSIS6* and *ARGINYL-TRNA:PROTEIN ARGINYLTRANSFERASE* [70]. The data represented herein was expressed as % fresh weight and minor changes in lipid content may be attributed to differences in the water content between *cca1lhy* and WS. For example, lower amounts of 30∶0-CoA in the *cca1lhy* mutant may suggest lower epidermal waxes and higher water loss in the mutant.

Variation in TAG content between CCA1-OX and Col-0 exists in dry seeds (Figure 8A) and was observed in 2- to 4-day-old seedlings (Figure 8C–E), but no significant differences were detected in 1- and 5-day-old seedlings (Figure 8B, F). It appears that differences in 18∶2 and 18∶3 FAs between dry seeds of CCA1-OX and Col-0 (Figure 10A) attributed to the TAG content increases in 2- to 4-day-old seedlings (Figure 8C–E) with 18∶2 being the major FA present (Figure 10). Meanwhile, a general elevation in the expression of β-oxidation key enzymes (*ACX1*, *ACX2*, and *KAT2*) in 5-day-old CCA1-OX (Figure 4), suggests that most acyl-CoA utilization correspondingly increased, concomitant with a decrease in many acyl-CoA species in CCA1-OX. Only 24∶0 and 30∶0-CoAs accumulated in CCA1-OX, perhaps indicative that other proteins are involved in lipid transport during storage reserve mobilization (Figure S4). Given the predominance of 24∶0 and 30∶0 acyl chains in the formation of sphingolipids and waxes as observed for 24∶0 and 30∶0 derivatives in the wax-deficient *eceriferum* mutants [92]–[96], the major changes noted in 24∶0 and 30∶0-CoAs in CCA1-OX may have contributed to the high levels of C24-OH in the formation of sphingolipids and waxes. Interestingly, it has been reported that *KCS16* and *SLD2* are transcriptionally regulated by the biological clock [53], and the extent of diurnal regulation in wax and sphingolipid biosynthesis remains to be further determined.

## Supporting Information

Figure S1 **Expression of** ***TOC1*** **and** ***GI*** **in 2- and 5-day-old seedlings germinated under 12-h-light/12-h-dark cycles.** (A) Comparison in expression between *TOC1* and *GI* in the *cca1lhy* mutant (closed rhombus) and wild-type WS (open circle) as investigated by qRT-PCR. (B) Comparison in expression between *TOC1* and *GI* in CCA1-OX (closed rhombus) and wild-type Col-0 (open circle) as investigated by qRT-PCR. Relative gene expression level on the Y axis was normalized against *IPP2*. Each time point represents a mean value of six repeats from two independent biological samples ± SE. White boxes, subjective day; black boxes, subjective night.
(TIF)

Figure S2 **The expression pattern of** ***ACBPs*** **and lipid metabolism genes in 9-day-old Arabidopsis seedlings.** The expression pattern was sieved out from the normalized CCEE dataset from the Additional Data File 1 of Covington et al. (2008). White boxes, subjective day; black boxes, subjective night.
(TIF)

Figure S3 **Germination frequencies of WS and the** ***cca1lhy*** **mutant under 12-h-light/12-h-dark cycles on half-strength MS medium supplemented with 20 mM sucrose.** (A) Freshly-harvested seeds of the *cca1lhy* mutant (closed rhombus) and wild-type WS (open circle). (B) After-ripening seeds of the *cca1lhy* mutant (closed rhombus) and wild-type WS (open circle) were harvested 3–6 months prior to the assay. Values are mean ± SD of measurements made on four separate batches of 50–100 seeds. Student's *t* test for ☆, P<0.01; ★, P<0.001.
(TIF)

Figure S4 **Acyl-CoA profiling of the** ***cca1lhy*** **mutant and CCA1-OX in comparison to wild-type Arabidopsis.** Acyl-CoA content of 5-day-old seedlings from the *cca1lhy* mutant, CCA1-OX, WS and Col-0 germinated under 12-h-light/12-h-dark cycles. White bar, wild-type WS; light gray bar, the *cca1lhy* mutant; dark gray bar, wild-type Col-0; black bar, CCA1-OX. n = 24; average ± SE. Student's *t* test for **, P<0.01; ***, P<0.001.
(TIF)

Figure S5 **Diurnal regulation of the major lipid metabolic pathways in germinating Arabidopsis seedlings.** Target genes in acyl-lipid transfer (*ACBP1*, *ACBP2*, *ACBP3*, *ACBP4*, *ACBP5* and *ACBP6*), lipolysis (*SDP1*), β-oxidation (*CTS*, *LACS6*, *LACS7*, *ACX1*, *ACX2*, *MFP2* and *KAT2*) and TAG synthesis (*DGAT1*, *DGAT2*, *DGAT3* and *PDAT1*) at Day 2 (A) and Day 5 (B) are represented in italics. Genes which displayed a 2-fold or greater value at peak expression over its lowest expression level in wild-type WS or Col-0, in both biological repeats, were deemed to be diurnally regulated. Genes which showed diurnal regulation in wild-type WS in qRT-PCR are coloured in orange; those diurnally-regulated in wild-type Col-0 are in blue; and those diurnally-regulated in both WS and Col-0 are in green.
(TIF)

Table S1 **Gene-specific primers for qRT-PCR used in this study.**
(DOC)

Table S2 **The expressed and circadian pattern of lipid metabolism genes in 9-day-old Arabidopsis seedlings mined from circadian microarray data sets.** Expressed and circadian pattern of lipid metabolism genes sieved out from Additional Data File 2 in Covington et al. (2008). Exp represents expressed; Cir represents circadian.
(DOC)
