# Supplementary figures and images for: Gene Expression in Plant Lipid Metabolism in Arabidopsis Seedlings
Source: PLoS One. 2014 Sep 29;9(9):e107372. doi: 10.1371/journal.pone.0107372 (PMC4180049; doi:10.1371/journal.pone.0107372)

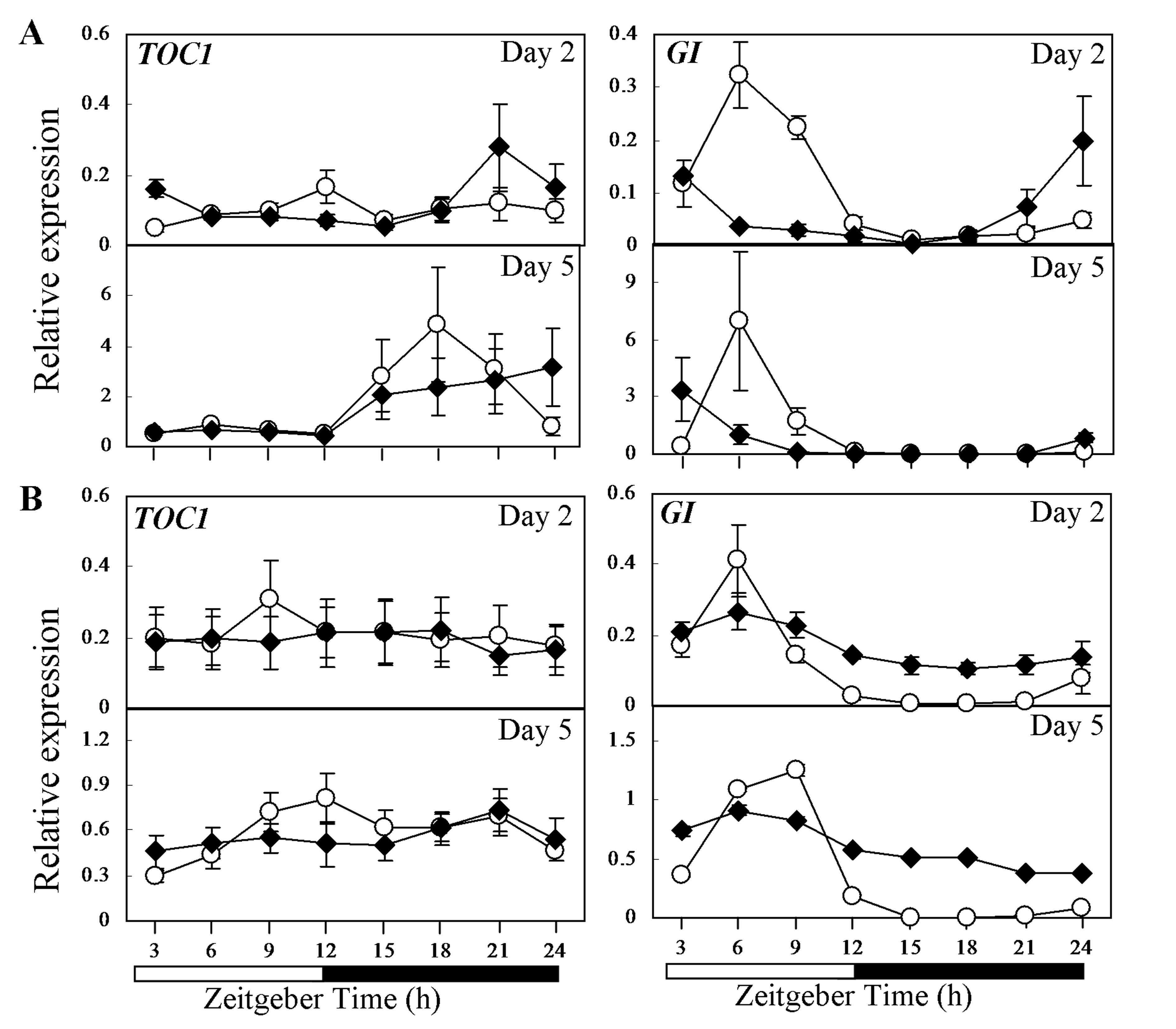

Supplement: Figure S1 — Expression of TOC1 and GI in 2- and 5-day-old seedlings germinated under 12-h-light/12-h-dark cycles. (A) Comparison in expression between TOC1 and GI in the cca1lhy mutant (closed rhombus) and wild-type WS (open circle) as investigated by qRT-PCR. (B) Comparison in expression between TOC1 and GI in CCA1-OX (closed rhombus) and wild-type Col-0 (open circle) as investigated by qRT-PCR. Relative gene expression level on the Y axis was normalized against IPP2. Each time point represents a mean value of six repeats from two independent biological samples ± SE. White boxes, subjective day; black boxes, subjective night. (TIF) [file pone.0107372.s001.tif]

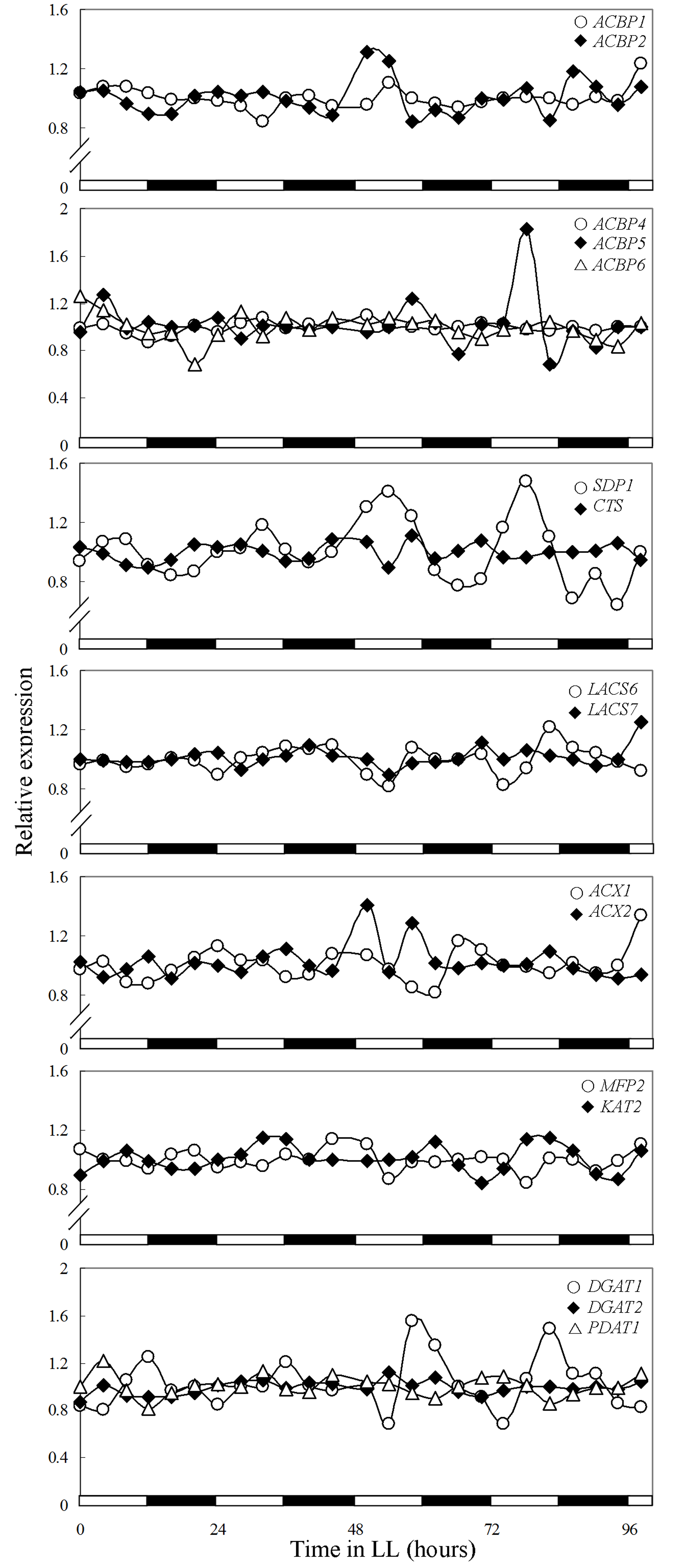

Supplement: Figure S2 — The expression pattern of ACBPs and lipid metabolism genes in 9-day-old Arabidopsis seedlings. The expression pattern was sieved out from the normalized CCEE dataset from the Additional Data File 1 of Covington et al. (2008). White boxes, subjective day; black boxes, subjective night. (TIF) [file pone.0107372.s002.tif]

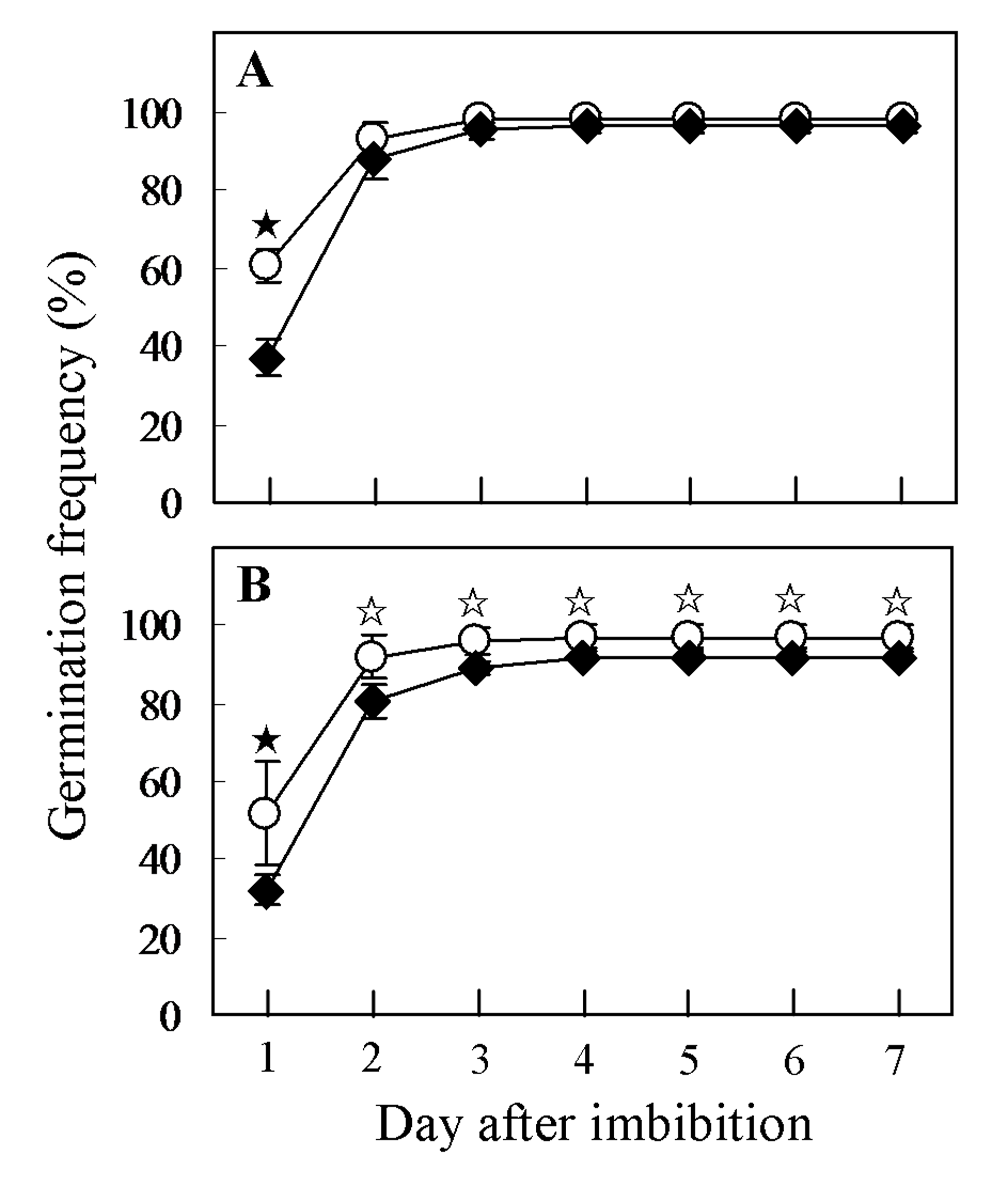

Supplement: Figure S3 — Germination frequencies of WS and the cca1lhy mutant under 12-h-light/12-h-dark cycles on half-strength MS medium supplemented with 20 mM sucrose. (A) Freshly-harvested seeds of the cca1lhy mutant (closed rhombus) and wild-type WS (open circle). (B) After-ripening seeds of the cca1lhy mutant (closed rhombus) and wild-type WS (open circle) were harvested 3–6 months prior to the assay. Values are mean ± SD of measurements made on four separate batches of 50–100 seeds. Student's t test for ☆, P<0.01; ★, P<0.001. (TIF) [file pone.0107372.s003.tif]

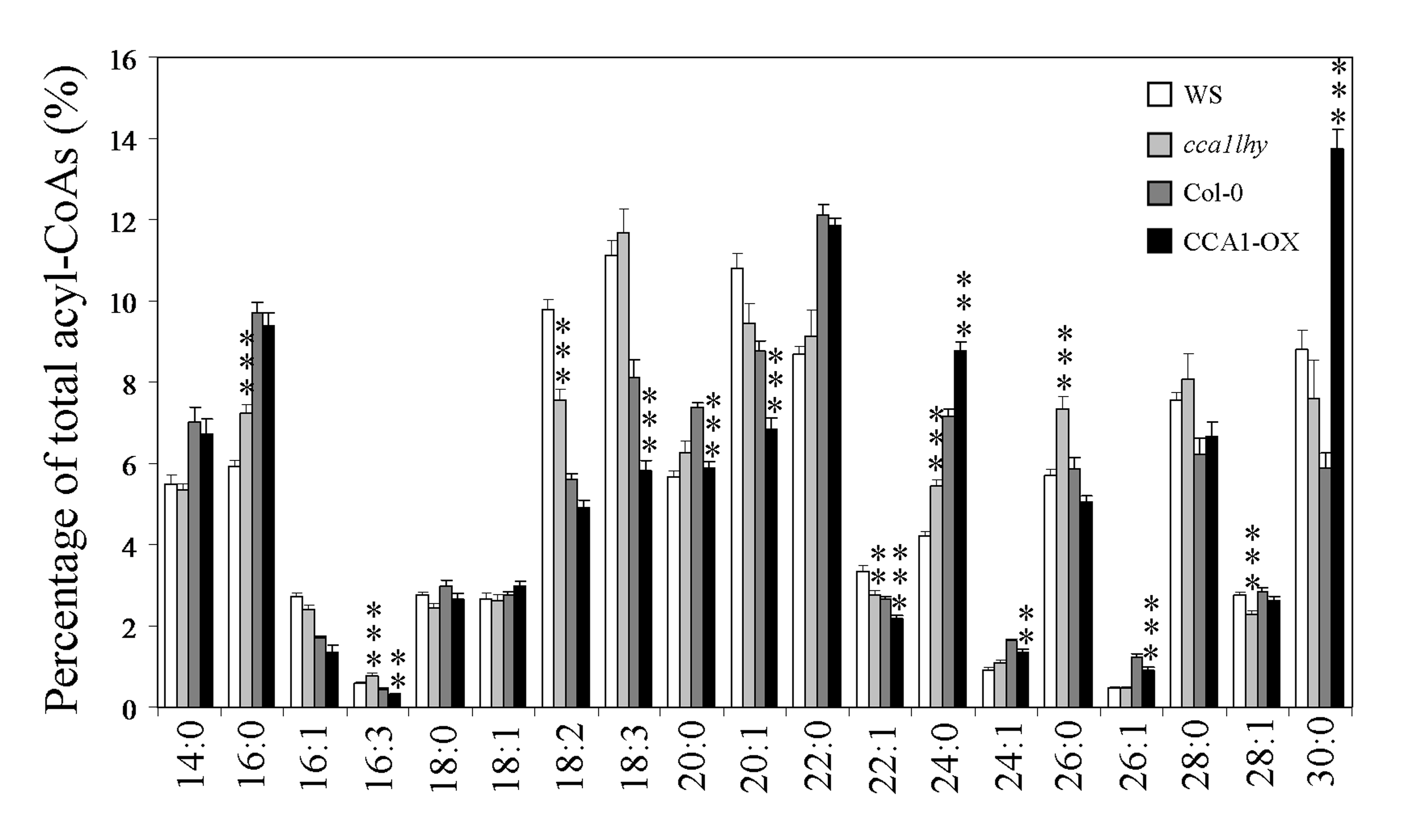

Supplement: Figure S4 — Acyl-CoA profiling of the cca1lhy mutant and CCA1-OX in comparison to wild-type Arabidopsis. Acyl-CoA content of 5-day-old seedlings from the cca1lhy mutant, CCA1-OX, WS and Col-0 germinated under 12-h-light/12-h-dark cycles. White bar, wild-type WS; light gray bar, the cca1lhy mutant; dark gray bar, wild-type Col-0; black bar, CCA1-OX. n = 24; average ± SE. Student's t test for **, P<0.01; ***, P<0.001. (TIF) [file pone.0107372.s004.tif]

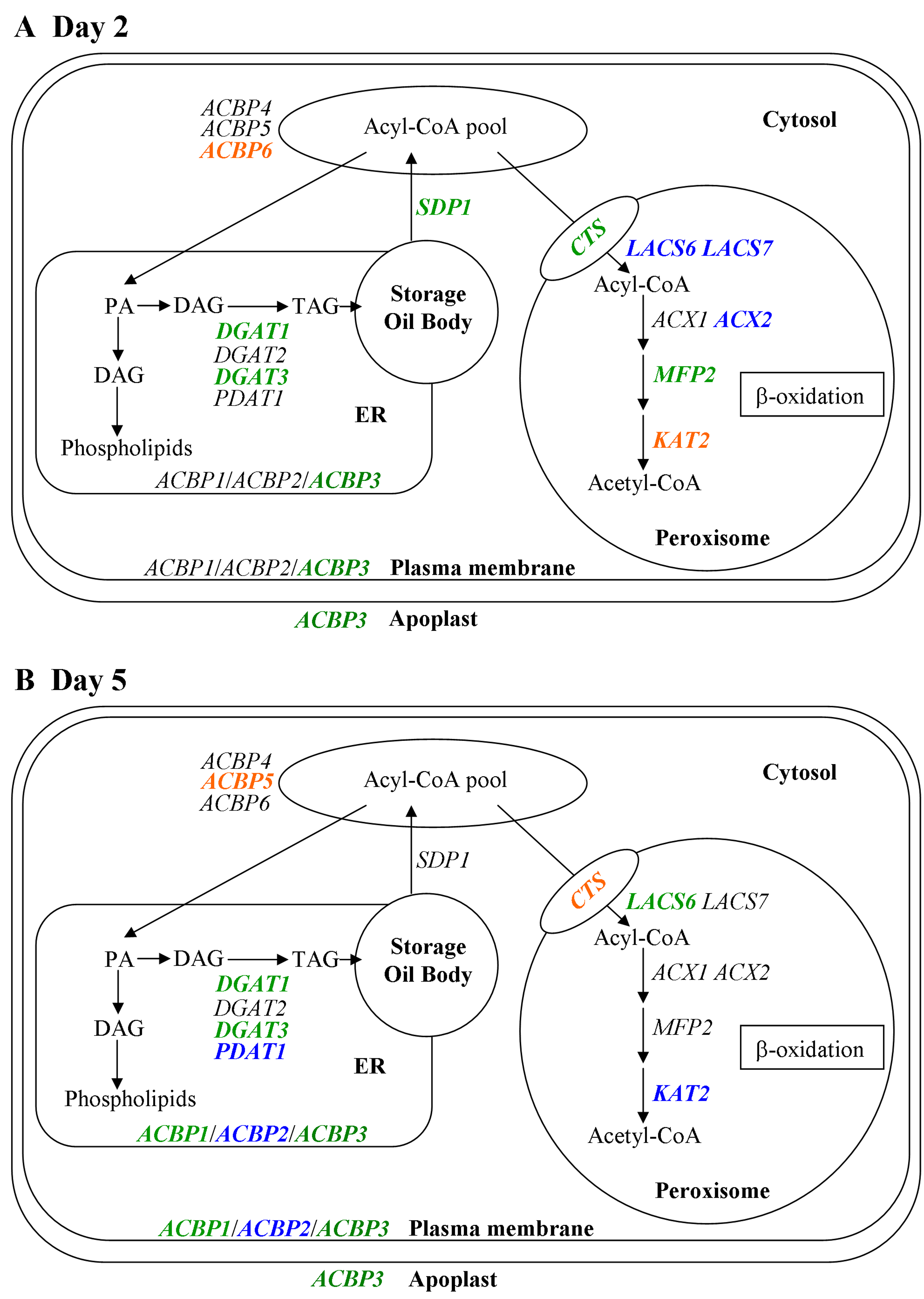

Supplement: Figure S5 — Diurnal regulation of the major lipid metabolic pathways in germinating Arabidopsis seedlings. Target genes in acyl-lipid transfer (ACBP1, ACBP2, ACBP3, ACBP4, ACBP5 and ACBP6), lipolysis (SDP1), β-oxidation (CTS, LACS6, LACS7, ACX1, ACX2, MFP2 and KAT2) and TAG synthesis (DGAT1, DGAT2, DGAT3 and PDAT1) at Day 2 (A) and Day 5 (B) are represented in italics. Genes which displayed a 2-fold or greater value at peak expression over its lowest expression level in wild-type WS or Col-0, in both biological repeats, were deemed to be diurnally regulated. Genes which showed diurnal regulation in wild-type WS in qRT-PCR are coloured in orange; those diurnally-regulated in wild-type Col-0 are in blue; and those diurnally-regulated in both WS and Col-0 are in green. (TIF) [file pone.0107372.s005.tif]
