# Supplementary material for: Gene Expression in Plant Lipid Metabolism in Arabidopsis Seedlings
Source: PLoS One. 2014 Sep 29;9(9):e107372. doi: 10.1371/journal.pone.0107372 (PMC4180049; doi:10.1371/journal.pone.0107372)
Supplement: Table S1 — Gene-specific primers for qRT-PCR used in this study. (DOC) [file pone.0107372.s006.doc]

**Table S1. Gene-specific primers for qRT-PCR used in this study**.

| **Primer (*gene*)** | **Sequence** |
| --- | --- |
| ML2410 (*TOC1*-F) | 5’-GGCTCACATCATCTTCATCGGGGT-3’ |
| ML2411 (*TOC1*-R) | 5’-TCCTACCATGAGATTCCATTGATGTG-3’ |
| ML2408 (*GI*-F) | 5’-TGAGAGATGGATCGATGGTCTTCAG-3’ |
| ML2409 (*GI*-R) | 5’-AGTCGCTTCTCAGTTGATGGATACT-3’ |
| ML1120 (*ACBP1*-F) | 5’-TGGAGATGCGTTATTGTGA-3’ |
| ML1121 (*ACBP1*-R) | 5’-GCGAGAAGGTAAGCGAAG-3’ |
| ML1122 (*ACBP2*-F) | 5’-GTGAGGCGGATTCGCTTGT-3’ |
| ML1123 (*ACBP2*-R) | 5’-TGCGGCGGCGGTAGTC-3’ |
| ML1202 (*ACBP3*-F) | 5’-GACAGGACGAACAGAGCG-3’ |
| ML1203 (*ACBP3*-R) | 5’-CAGATTCAGCGACTAAGACAT-3’ |
| ML1238 (*ACBP4*-F) | 5’-TGGCGGTGATAACAAG-3’ |
| ML1239 (*ACBP4*-R) | 5’-GAAAGACTACCTGGCAAA-3’ |
| ML1206 (*ACBP5*-F) | 5’-CCGACCGCATTATCACA-3’ |
| ML1207 (*ACBP5*-R) | 5’-CAAGTTGGTTCCGCACA-3’ |
| ML1208 (*ACBP6*-F) | 5’-TGTCCTCGTCTTCTCCG-3’ |
| ML1209 (*ACBP6*-R) | 5’-CAGCGTGCTCCTCAAA-3’ |
| ML1913 (*SDP1*-F) | 5’-TGCTCAGGAGTGGGAAGGTGATG-3’ |
| ML1914 (*SDP1*-R) | 5’-GAGAGCTTCTCCCAAGTGCATCTTC-3’ |
| ML1863 (*CTS*-F) | 5’-TTGCCGCTAAGGTTCGAGCTATG-3’ |
| ML1864 (*CTS*-R) | 5’-AGCATCCGTCAGAAGGGCAGAGT-3’ |
| ML1865 (*LACS6*-F) | 5’-GGTGCTTCACCTCTCTCTCCTGAAG-3’ |
| ML1866 (*LACS6*-R) | 5’-GAGGTTATCACCCTCGTCCATTCC-3’ |
| ML1871 (*LACS7*-F) | 5’-GGTGTTGTGTTGACTCATGGAAACTTG-3’ |
| ML1872 (*LACS7*-R) | 5’-GAAACCGACAGCAACACCACCATAC-3’ |
| ML1873 (*ACX1*-F) | 5’-CACATAATGGTGGCATTGAGACACA-3’ |
| ML1874 (*ACX1*-R) | 5’-TCACTAGCCGCCAGTCTTTCAGTTAC-3’ |
| ML1867 (*ACX2*-F) | 5’-GGGATTAAGATGGGCGTTCAATACAG-3’ |
| ML1868 (*ACX2*-R) | 5’-GTGTAATTCAGTCATGGCAAAGCAAC-3’ |
| ML1875 (*MFP2*-F) | 5’-GTCGCTGCCATTGATGGACTTG-3’ |
| ML1876 (*MFP2*-R) | 5’-GGGCTTTGGTGAGACCAACAAGAC-3’ |
| ML1877 (*KAT2*-F) | 5’-AGAAGGATGGCACCACTACTGCTG-3’ |
| ML1878 (*KAT2*-R) | 5’-GATTGCAGGGTCAACACCAACTG-3’ |
| ML2026 (*DGAT1*-F) | 5’-GGAGGGCGAGAGAGAGTCCACTT-3’ |
| ML2027 (*DGAT1*-R) | 5’-CCAATCTCGCAGCGATCTTGAAC-3’ |
| ML2028 (*DGAT2*-F) | 5’-CCGAGCTGAGGAACATTCAAATCA-3’ |
| ML2029 (*DGAT2*-R) | 5’-CGACCATATTTGCTACGATGATCGA-3’ |
| ML2030 (*DGAT3*-F) | 5’-GGTCTTGATCCTGAAGCTGGTTTG-3’ |
| ML2031 (*DGAT3*-R) | 5’-TCAGTCATCTTCTTCATGGCTTTGG-3’ |
| ML2032 (*PDAT1*-F) | 5’-TGCTGAAGCAAAGGATGTTGCA-3’ |
| ML2033 (*PDAT1*-R) | 5’-CCTTTCTCCGGTGACCAATCAAG-3’ |
| *IPP2*-F | 5’-GTATGAGTTGCTTCTCCAGCAAAG-3’ |
| *IPP2*-R | 5’-GAGGATGGCTGCAACAAGTGT-3’ |

F represents Forward primer

R represents Reverse primer
