# Supplementary material for: Gene Expression in Plant Lipid Metabolism in Arabidopsis Seedlings
Source: PLoS One. 2014 Sep 29;9(9):e107372. doi: 10.1371/journal.pone.0107372 (PMC4180049; doi:10.1371/journal.pone.0107372)
Supplement: Table S2 — The expressed and circadian pattern of lipid metabolism genes in 9-day-old Arabidopsis seedlings mined from circadian microarray data sets. Expressed and circadian pattern of lipid metabolism genes sieved out from Additional Data File 2 in Covington et al. (2008). Exp represents expressed; Cir represents circadian. (DOC) [file pone.0107372.s007.doc]

**Table S2. The expressed and circadian pattern of lipid metabolism genes in 9-day-old Arabidopsis seedlings mined from circadian microarray data sets.**

|  | Identifiers | | Covington | | Edwards | | CCEE | | EECC | | CECE | | C+E intersection | | C+E union | | Michael 1 | | Michael 2 | |
| --- | --- | --- | --- | --- | --- | --- | --- | --- | --- | --- | --- | --- | --- | --- | --- | --- | --- | --- | --- | --- |
| *GENE* | AFFY | AGI | Exp | Cir | Exp | Cir | Exp | Cir | Exp | Cir | Exp | Cir | Exp | Cir | Exp | Cir | Exp | Cir | Exp | Cir |
| *ACBP1* | 248269_at | AT5G53470 | 1 |  | 1 |  | 1 |  | 1 |  | 1 |  | 1 |  | 1 |  | 1 |  | 1 |  |
| *ACBP2* | 253840_at | AT4G27780 | 1 |  | 1 |  | 1 |  | 1 |  | 1 |  | 1 |  | 1 |  | 1 |  | 1 |  |
| *ACBP3* |  | AT4G24230 | data not available | | | | | | | | | | | | | | | | | |
| *ACBP4* | 259159_at | AT3G05420 | 1 |  | 1 | 1 | 1 |  | 1 |  | 1 |  | 1 |  | 1 |  | 1 |  | 1 |  |
| *ACBP5* | 246731_at | AT5G27630 | 1 |  | 1 |  | 1 |  | 1 |  | 1 |  | 1 |  | 1 |  | 1 |  |  |  |
| *ACBP6* | 246267_at | AT1G31812 | 1 |  | 1 |  | 1 |  | 1 |  | 1 |  | 1 |  | 1 |  | 1 |  | 1 | 1 |
| *SDP1* | 250877_at | AT5G04040 | 1 | 1 | 1 | 1 | 1 | 1 | 1 | 1 | 1 | 1 | 1 | 1 | 1 | 1 | 1 | 1 | 1 | 1 |
| *CTS* | 252830_at | AT4G39850 | 1 | 1 | 1 |  | 1 |  | 1 |  | 1 |  | 1 |  | 1 |  | 1 |  | 1 |  |
| *LACS6* | 258563_at | AT3G05970 | 1 |  | 1 |  | 1 | 1 | 1 | 1 | 1 | 1 | 1 | 1 | 1 | 1 | 1 | 1 | 1 | 1 |
| *LACS7* | 246789_at | AT5G27600 | 1 |  | 1 |  | 1 |  | 1 |  | 1 |  | 1 |  | 1 |  | 1 |  | 1 |  |
| *ACX1* | 245249_at | AT4G16760 | 1 | 1 | 1 |  | 1 | 1 | 1 | 1 | 1 | 1 | 1 | 1 | 1 | 1 | 1 | 1 | 1 | 1 |
| *ACX2* | 247176_at | AT5G65110 | 1 |  | 1 |  | 1 |  | 1 |  | 1 |  | 1 |  | 1 |  | 1 | 1 | 1 | 1 |
| *MFP2* | 258555_at | AT3G06860 | 1 |  | 1 |  | 1 |  | 1 |  | 1 |  | 1 |  | 1 |  | 1 |  | 1 |  |
| *KAT2* | 245168_at | AT2G33150 | 1 |  | 1 | 1 | 1 | 1 | 1 | 1 | 1 | 1 | 1 | 1 | 1 | 1 | 1 | 1 | 1 | 1 |
| *DGAT1* | 267280_at | AT2G19450 | 1 | 1 | 1 | 1 | 1 | 1 | 1 | 1 | 1 | 1 | 1 | 1 | 1 | 1 | 1 | 1 | 1 | 1 |
| *DGAT2* | 252064_at | AT3G51520 | 1 |  | 1 |  | 1 |  | 1 |  | 1 |  | 1 |  | 1 |  | 1 |  | 1 |  |
| *DGAT3* |  | AT1G48300 | data not available | | | | | | | | | | | | | | | | | |
| *PDAT1* | 250253_at | AT5G13640 | 1 |  | 1 | 1 | 1 | 1 | 1 | 1 | 1 | 1 | 1 | 1 | 1 | 1 | 1 |  | 1 |  |

Expressed and circadian pattern of lipid metabolism genes sieved out from Additional Data File 2 in Covington et al. (2008). Exp represents expressed; Cir represents circadian.
